# Specificity of a β-porphyranase produced by the carrageenophyte red alga *Chondrus crispus* and implications of this unexpected activity on red algal biology

**DOI:** 10.1016/j.jbc.2022.102707

**Published:** 2022-11-17

**Authors:** Guillaume Manat, Mathieu Fanuel, Diane Jouanneau, Murielle Jam, Jessica Mac-Bear, Hélène Rogniaux, Théo Mora, Robert Larocque, Agnieszka Lipinska, Mirjam Czjzek, David Ropartz, Elizabeth Ficko-Blean

**Affiliations:** 1 CNRS, Integrative Biology of Marine Models (LBI2M), Station Biologique de Roscoff (SBR), Sorbonne Université, Roscoff, France; 4 CNRS, FR 2424, Station Biologique de Roscoff, Sorbonne Université, Roscoff, France; 2 INRAE, UR BIA, Nantes, France; 3 INRAE, BIBS Facility, Nantes, France

**Keywords:** red algae, *Chondrus crispus*, porphyran, carrageenan, porphyranase, family 16 glycoside hydrolase, GH16, sulfated polysaccharides, extracellular matrix, cell wall, cDNA, complementary DNA, CTD, charge transfer dissociation, ECM, extracellular matrix, ESI, electrospray ionization, FACE, fluorophore-assisted carbohydrate electrophoresis, MALS, multiangle light scattering, MS, mass spectrometry, MW, molecular weight, SEC, size-exclusion chromatography, UHPLC, ultra-HPLC

## Abstract

The carrageenophyte red alga *Chondrus crispus* produces three family 16 glycoside hydrolases (CcGH16-1, CcGH16-2, and CcGH16-3). Phylogenetically, the red algal GH16 members are closely related to bacterial GH16 homologs from subfamilies 13 and 14, which have characterized marine bacterial β-carrageenase and β-porphyranase activities, respectively, yet the functions of these CcGH16 hydrolases have not been determined. Here, we first confirmed the gene locus of the *ccgh16-3* gene in the alga to facilitate further investigation. Next, our biochemical characterization of CcGH16-3 revealed an unexpected β-porphyranase activity, since porphyran is not a known component of the *C. crispus* extracellular matrix. Kinetic characterization was undertaken on natural porphyran substrate with an experimentally determined molecular weight. We found CcGH16-3 has a pH optimum between 7.5 and 8.0; however, it exhibits reasonably stable activity over a large pH range (pH 7.0–9.0). CcGH16-3 has a *K*_*M*_ of 4.0 ± 0.8 μM, a *k*_cat_ of 79.9 ± 6.9 s^−1^, and a *k*_cat_/*K*_*M*_ of 20.1 ± 1.7 μM^−1^ s^−1^. We structurally examined fine enzymatic specificity by performing a subsite dissection. CcGH16-3 has a strict requirement for D-galactose and L-galactose-6-sulfate in its −1 and +1 subsites, respectively, whereas the outer subsites are less restrictive. CcGH16-3 is one of a handful of algal enzymes characterized with a specificity for a polysaccharide unknown to be found in their own extracellular matrix. This β-porphyranase activity in a carrageenophyte red alga may provide defense against red algal pathogens or provide a competitive advantage in niche colonization.

The macroalgal extracellular matrix (ECM) supports many functions such as protection against osmotic stress and desiccation, macroalgal flexibility, heavy metals bioabsorption, and is a barrier against pathogens (1, 2). The major components of macroalgal ECMs are polysaccharides, which can have a large diversity of structures, even in the same species. Although algal polysaccharides have had major advances in their structural characterization, little data are available about macroalgal ECM interactions including biosynthesis, modification, and recycling. Most studies characterizing macroalgal ECM interactions have used WT enzymes. Two enzymatic activities were characterized in multicellular green algae, namely xyloglucan:xyloglucan endotransglucosylase (XET) and mixed-linkage-glucan:xyloglucan endotransglucosylase (MXE), activities that function in cell wall extension and remodeling (3, 4, 5). A mannuronan C5-epimerase activity was demonstrated from protoplasts of the brown macroalga *Laminaria digitata* (6). In red macroalgae, galactose-sulfurylase enzymes catalyze the elimination of sulfate from galactose-6-sulfate or galactose-2,6-sulfate to form the 3,6-anhydro bridge (7). Galactose-sulfurylase activity was first demonstrated on porphyran (a precursor for agar) using enzyme extracted from *Porphyra umbilicalis* (8). Two galactose-sulfurylase enzymes were purified from *Chondrus crispus*, galactose-sulfurylase I and galactose-sulfurylase II, and subsequently characterized as forming the 3,6-anhydro-d-galactose moiety in carrageenan (9). Even fewer biochemical studies have been carried out on recombinant macroalgal ECM-active enzymes; this is presumably because protein production is challenging and the acidically charged glycan substrates are highly complex. Nevertheless, a recombinant brown algal mannuronan C5-epimerase, involved in alginate biosynthesis, was successfully refolded to produce active enzyme (10). In addition, two alginate lyases have been biochemically characterized, one from a red macroalga (11), where alginate is not a known component, and the other from a brown macroalga (12).

*C. crispus* is a carrageenophyte red macroalgae (Rhodophyta, Gigartinales) found along the northern Atlantic coast (Europe and America) that is mainly used in food and cosmetic industry for the properties of its algal ECM (13). The *C. crispus* ECM contains carrageenans that are linear, sulfated galactans with hybrid compositions; the basic unit is a D-galactose (G) disaccharide with alternating β-1,4 and α-1,3 linkages that is modified by sulfations on the disaccharide unit (14). Another important modification is the unique bicyclic sugar α-3,6-anhydro-D-galactose (DA) (14). Its biosynthesis was characterized using WT galactose-sulfurylases, the enzymatic desulfation of the C6 hydroxyl leads to the formation of the 3,6-anhydro-bridge (9, 15).

*C. crispus* has a complex isomorphic haplodiplontic life cycle. In gametophytes (n), the major carrageenan structures are the κ and ι-carrageenans and the minor biosynthetic precursors are μ- and ν-carrageenans (16, 17). In tetrasporophytes (2n), there is only λ-carrageenan that has been described in the literature (18). The gametophyte and tetrasporophyte life stages of *C. crispus* are isomorphic in the absence of reproductive structures; this suggests that the carrageenan structure may be important for the physiological differentiation between life stages of *C. crispus* (19). This is supported by the susceptibility of tetrasporophytes, but not gametophytes, of *C. crispus* to a green algal pathogen, *Ulvella (Acrochaete) operculata* (20, 21). When artificially introduced to the milieu, λ-carrageenan oligosaccharides induced increased virulence toward gametophytes and κ-carrageenan oligosaccharides reduced virulence toward tetrasporopytes by the green algal pathogen (22). Thus, life cycle specific carrageenan metabolites have functionally distinct biological signaling properties and influence susceptibility to *U. operculata*.

The genome sequencing and annotation of *C. crispus* has led to the identification of three genes (*ccgh16-1*, *ccgh16-2*, and *ccgh16-3*) coding for CAZy (Carbohydrate Active Enzymes database, URL http://www.cazy.org/) GH16 family members (CcGH16-1, CcGH16-2, and CcGH16-3, Fig. 1 and Table S1) (23, 24). The differences in carrageenan composition between the isomorphic life stages of *C. crispus* suggest that genes involved in carrageenan biosynthesis may be differentially expressed between these life stages. The gene *ccgh16-1* was silent in the tetrasporophyte and gametophyte stages under the conditions assayed. On the other hand, the genes cc*gh16*-*2* and cc*gh16-3* were expressed in all the life cycle stages, with a significant increase of cc*gh16-3* expression in male gametophytes and tetrasporophytes relative to the female gametophytes (19).

**Figure 1:**
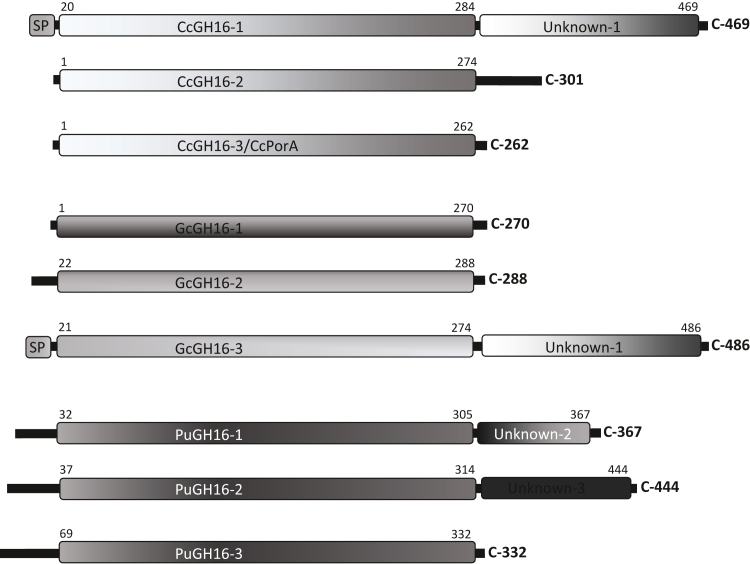
**Modular structure of red algal GH16 enzymes.** Cc is *Chondrus crispus*, Gc is *Gracilariopsis chorda*, and Pu is *Porphyra umbilicalis*. SP represents a putative signal peptide.

Two variants of ‘typical’ active sites within the GH16 family exist with the ExDxE and ExDxxE conserved amino acid motifs that are clearly phylogenetically separated (25, 26, 27). In the first motif, the hyaluronidase (28), laminarinase (29), β-1,3-galactanase (30), β-1,3/β-1,4-glucanase (31, 32), and xyloglucan endotransglucosylase/hydrolase (33) activities are defined. The second motif is possessed by enzymes from several bacteria that degrade the ECMs of red algae including β-agarases (34, 35), β-porphyranases (36), and κ- or β-carrageenanases (all β-1,4-galactanases) (37, 38, 39). All of the *C. crispus* GH16 enzymes belong to the second, ExDxxE clade shared by GH16 members from both carrageenophyte and agarophyte red algae (25, 26, 27). The similarity was supported by a protein sequence and structure similarity network analysis (25) and phylogenetic analyses of the GH16 family (26). These studies revealed that the *C. crispus* GH16 enzymes form a subclade together with the red algal GH16 enzymes from *P. umbilicalis*. Moreover, close neighboring clades were shown to contain biochemically characterized bacterial β-porphyranases and β-agarases (GH16 subfamilies 11, 12, 13, 14, 16, and 26) (25, 26).

It was initially hypothesized that the three CcGH16 proteins identified may be involved in carrageenan biosynthesis, remodeling, and/or recycling, based on the carrageenan composition of the ECM of *C. crispus* and the CcGH16 enzyme’s phylogenetic relationship with marine bacterial sulfated polysaccharide degrading enzymes (19). Here, we investigate the biochemistry of the red algal CcGH16-3 enzyme, which was found to have an unexpected β-porphyranase activity. This investigation provides one of only a couple biochemical studies on recombinant red algal glycoside hydrolases and brings a new perspective to glycoside hydrolase function in seaweed.

## Results and discussion

In the carrageenophyte red alga *C. crispus*, three different CcGH16 enzymes (1 - CDF40276.1, 2 - CDF41280.1, and 3 - CDF33251.1) were identified with 469 (52.6 kDa), 301 (35.8 kDa), and 262 (29.9 kDa) amino acids, respectively, and sharing between 30% and 34% sequence identity (Fig. 1). One GH16 gene in particular, *ccgh16-3*, was previously identified (19) as differentially regulated between the stages of the complex isomorphic haplodiplontic life cycle of *C. crispus* (40). CcGH16-3 amino acid BLAST revealed the closest GH16 homologs in other red algae were from agarophyte red algae (*Porphyra*, *Gracilariopsis*) with 33% to 48% sequence identity but this result could be explained by the weakness of the red algal genome database and the distinct lack of carrageenophyte red algal genomes (with the sole exception at the time of writing of *C. crispus*). Phylogenetic analyses revealed that the CcGH16 enzymes cluster closest first with uncharacterized marine red algal and then bacterial GH16 enzymes including β-porphyranases and β-agarases (26). Since porphyran is not a known component of the *C. crispus* ECM, this led to the hypothesis that the *C. crispus* GH16 enzymes are active as carrageenases and involved in ECM modification. Furthermore, the *ccgh16-3* gene from *C. crispus* demonstrates differential gene expression between tetrasporophytes relative to female gametophytes; these are multicellular life stages that have different carrageenan content (19). Overall, this suggested that the *C. crispus* CcGH16 enzymes may be involved in red algal sulfated ECM polysaccharide modification.

### Evidence the *ccgh16-3* gene is eukaryotic and not bacterial

**Figure 2:**
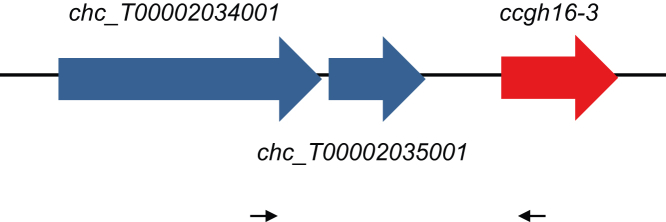
**Schematic representation of *ccgh16-3* genomic environment with primer positions represented by *black arrows*: *chc_t00002034001*-F and *genccgh16-3*-R (**Table S2**)**.

**Figure 3:**
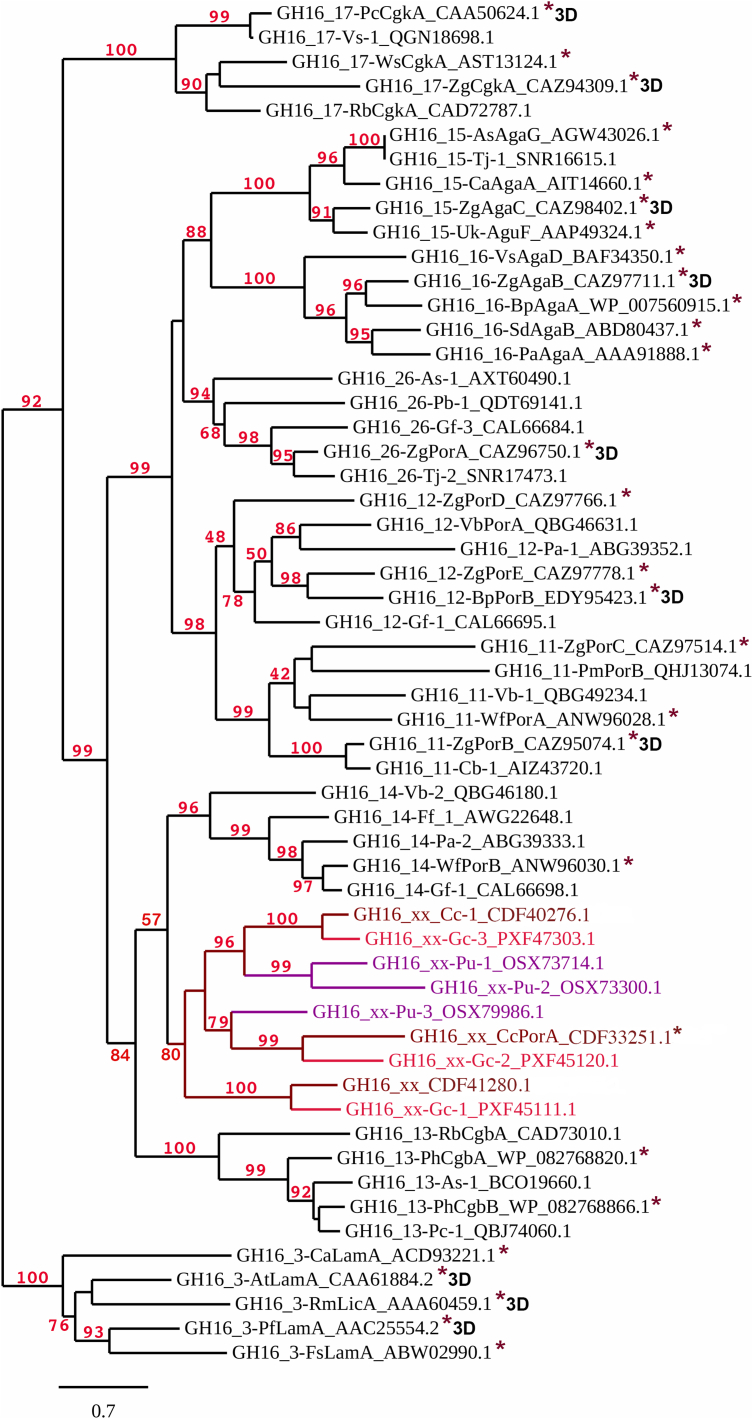
**Phylogenetic analysis of the red algal GH16 enzymes (GH16_xx) shown in different shades of *red* depending on the red algal species (**Table S1**).** Only GH16 catalytic modules were included in the analysis, not full-length sequences (Supporting Information File 1). Nearest subfamilies included in the analysis were chosen based on their amino acid sequence similarity to the red algal GH16 enzymes. Bootstraps above 40 are shown. *Asterisks* (∗) show characterized GH16 enzymes in the CAZy database (23) and 3D indicates the structures have been determined. The more distantly related GH16 subfamily 3 laminarinases (GH16_3) were chosen as an outgroup.

The annotation of the genome of *C. crispus* initially identified the *gh16* genes in the alga (24). RNA-seq analysis further detected differential expression of the *ccgh16-3* gene (19). However, due to the close phylogenetic relationship to bacterial GH16 enzymes, we thought it to be prudent to confirm that the *ccgh16-3* gene is indeed located in the *C. crispus* genome and was not from bacterial contamination. In order to do so, we analyzed the context of the *ccgh16-3* genetic locus (Fig. 2) and undertook a fine GH16 subfamily phylogenetic analysis (Fig. 3).

Upstream of the *ccgh16-3* gene of *C. crispus*, the gene *chc_t00002034001* codes for a protein predicted by BLAST (41) to be the large subunit of the eukaryotic protein guanidine triphosphate phosphatase 1 (GTPase1). The closest identified non–red algal homologs of the GTPase1 protein are fungal: *Tuber magnatum* and *Aspergillus chevalieri*, which share 39.7% and 38.9% sequence identity over 79.0% query coverage. The closest characterized GTPase homologs are found in *Arabidopsis thaliana*, and in fungi, these GTPases are important for eukaryotic ribosomal biogenesis (42, 43). The *chc_t00002034001* gene is followed by *chc_t00002035001*, which has candidates in both bacteria and eukaryotes and is predicted to code for the redox protein peroxiredoxin. The *chc_t00002035001* gene is followed by the *ccgh16-3* gene (*chc_t00009578001*) (Fig. 2) (24). Using PCR, we were successfully able to amplify a 1.6 kb DNA fragment from *C. crispus* tetrasporophyte genomic DNA template located between the eukaryotic gene *chc_t00002034001* and *ccgh16-3* (Fig. 2). Sequencing confirmed the predicted sequences for the two target genes *chc_t00002034001* and *ccgh16-3*. This result confirms that the *ccgh16-3* gene is indeed found in the *C. crispus* genome and it further supports the genome assembly of *C. crispus* (24).

Red algal GH16 enzymes group together in a solid red algal clade (bootstrap 80) supporting the eukaryotic nature of the CcGH16 enzymes (Fig. 3). The closest phylogenetically related subfamilies are subfamilies 13 and 14, which include characterized marine bacterial β-carrageenase (38) and β-porphyranase (44) activities, respectively. Curiously, the red algal sequences are more distant to the classical bacterial κ-carrageenases, which is a highly divergent subfamily and has been identified to be the most distinct subfamily segregating at comparatively high E-value thresholds among those that are active on marine polysaccharides (25). This implies a convergent evolution toward the activity on carrageenan/porphyran and that GH16 enzymes with these activities have evolved at least two times from different bacterial origins. There are three distinct GH16 enzymes in *C. crispus*, which is reflected in *Gracilariopsis chorda* but not in *P. umbilicalis*. Thus, CcGH16-1 and GcGH16-3 form a clade with a bootstrap of 100, CcGH16-2 and GcGH16-1 form a clade with a bootstrap of 100, and CcGH16-3 and GcGH16-2 form a clade with a bootstrap of 99. Interestingly, the modular structures of CcGH16-1 and GcGH16-3 also correlate (Fig. 1). This suggests that the GH16 family in red algae resulted from a horizontal gene transfer (45) from a marine bacterium to a common red algal ancestor, subsequently followed by gene duplication events. The evolutionary divergence within the red algal GH16 enzymes could be reflective of differing specificities; however, there is a strong likelihood these specificities are related to the red algal ECM polysaccharides carrageenan and/or porphyran.

### Cloning and heterologous production of the *CcGH16-3* enzyme

A fragment of the *ccgh16-3* gene coding for the predicted GH16 catalytic module was amplified from complementary DNA (cDNA) produced from RNA extractions of local *C. crispus* tetrasporophytes and was cloned into the modified pET vector pRF3 (19, 46). The sequencing of the clones highlighted a few differences of the CcGH16-3 with the genome predicted amino acid sequence: L18P, P33L, and L226V mutations, which are not highly conserved residues in our GH16 alignment (Fig. S1), and these variant residues do occur in other porphyranase GH16 sequences. The mutations between the *C. crispus* genomic organism and *C. crispus* samples collected on the rocky shore could be explained by intraspecies genomic variations (47, 48).

Heterologous production of soluble CcGH16-3 was done in *Escherichia coli* BL21(DE3). Cobalt-NTA was used for purification by affinity chromatography and provided good protein purity as judged by SDS-PAGE (Fig. S2). The enzyme migrated as a single band of ∼30 kDa by SDS-PAGE that is coherent with a predicted size of 31.1 kDa. The gel filtration profile was composed of only one peak at approximatively 25 kDa, indicating that the enzyme is monomeric.

### Unexpected porphyranase activity discovered in a carrageenophyte red alga

**Figure 4:**
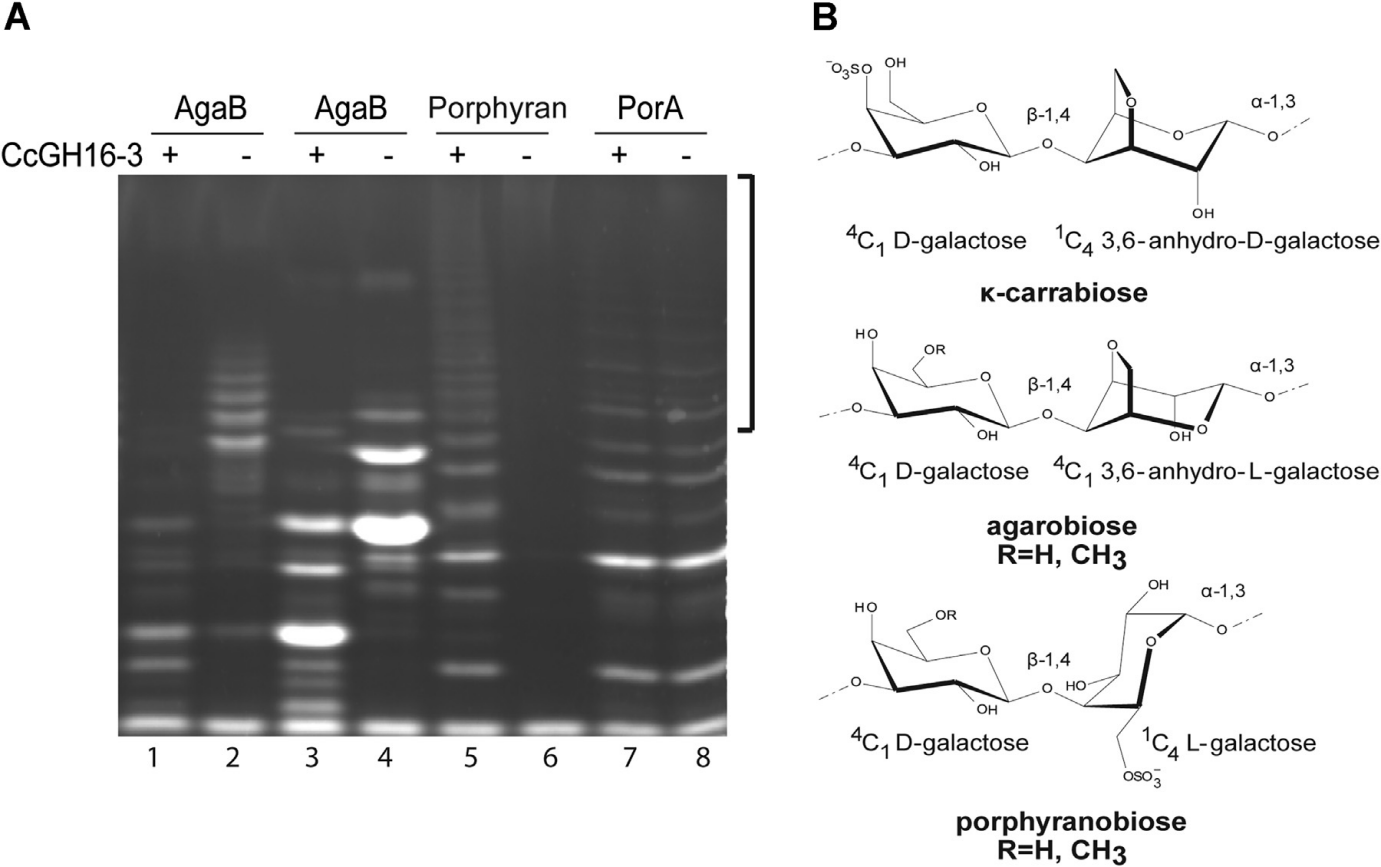
**Specificity of CcGH16-3 for porphyran.***A*, FACE (83) of hydrolysis products released by CcGH16-3 at 0.28 μM, demonstrating CcGH16-3 (+) is active on oligoporphyran digested by ZgAgaB but not with oligoporphyran digested by ZgPorA. Low MW ZgAgaB produced oligosaccharide substrate (F21-F22) is in lanes 1 and 2, high MW ZgAgaB produced oligosaccharide substrate (F5-F16) is in lanes 3 and 4, porphyran substrate is in lanes 5 and 6, and oligoporphyran substrate digested by ZgPorA is in lanes 7 and 8 (51). The negative control (−) was substrate with buffer A (10 mM Tris–HCl pH 8 and 100 mM NaCl). The higher MW region showing differences between CcGH16-3 (lane 5) and ZgPorA (lane 8) digestion on the porphyran polysaccharide is highlighted by a *black bracket*. Samples were labeled with the ANTS fluorophore and separated by PAGE. *B*, schematic representation of κ-carrabiose, agarobiose, and porphyranobiose motifs. FACE, fluorophore-assisted carbohydrate electrophoresis; MW, molecular weight.

To discriminate the substrates that could be hydrolyzed by CcGH16-3, different polysaccharides and oligosaccharides of carrageenans and agars were tested *in vitro* (34, 49, 50, 51) but no degradation of any carrageenans or agars was detected (data not shown). Unexpectedly, endolytic hydrolysis was confirmed by the laddered cleavage pattern on porphyran extracted from *Porphyra dioica* after treatment with CcGH16-3 (Fig. 4*A* lane 5–6). Porphyran (Fig. 4*B*) is a hybrid complex polysaccharide with both agarobiose and porphyranobiose motifs (as described for *P. umbilicalis*) that can be further modified with methyl (Me) groups (51, 52, 53, 54).

To understand whether CcGH16-3 was targeting porphyranobiose or agarobiose motifs, we also analyzed porphyran predigested by previously characterized GH16 members ZgAgaB or ZgPorA (34, 54) produced by the marine bacterium *Zobellia galactanivorans*. ZgAgaB is described as an enzyme active on hybrid substrate and most active on high agarose extracts. Its cleavage products are neo-series hybrid agarobiose and porphyranobiose containing oligosaccharides. Neo refers to oligosaccharides having the α-L-galactose-6-sulfate unit or the α-3,6-anhydro-L-galactose unit on the nonreducing end. ZgPorA hydrolyzes porphyranobiose motifs, leaving mainly neo-series agarobiose motifs. These enzymatically pretreated substrates were then digested with CcGH16-3 and the products of digestion were analyzed by fluorophore-assisted carbohydrate electrophoresis (FACE) (Fig. 4*A* lane 1–4, 7 and 8). The appearance of lower size bands with oligo-neoporphyran predigested by ZgAgaB, but not oligo-neoporphyran predigested by ZgPorA, was observed on the gel (Fig. 4*A*). The CcGH16-3 activity on the products of digestion of ZgAgaB and not ZgPorA suggested that CcGH16-3 is a porphyranase and thus in marine CAZyme nomenclature may also be referred to as CcPorA. FACE gel comparison between CcGH16-3 and ZgPorA established that the smaller oligosaccharides released migrated comparably with similar intensities for both enzymes; thus, these oligosaccharides are likely similar. The longer oligosaccharides seem to have more diversity in the CcGH16-3 degradation relative to ZgPorA (Fig. 4*A* comparing lanes 5 and 8), suggesting some subtle differences in substrate recognition between the two enzymes (36, 51).

### Biochemical characterization of *CcGH16-3*

**Figure 5:**
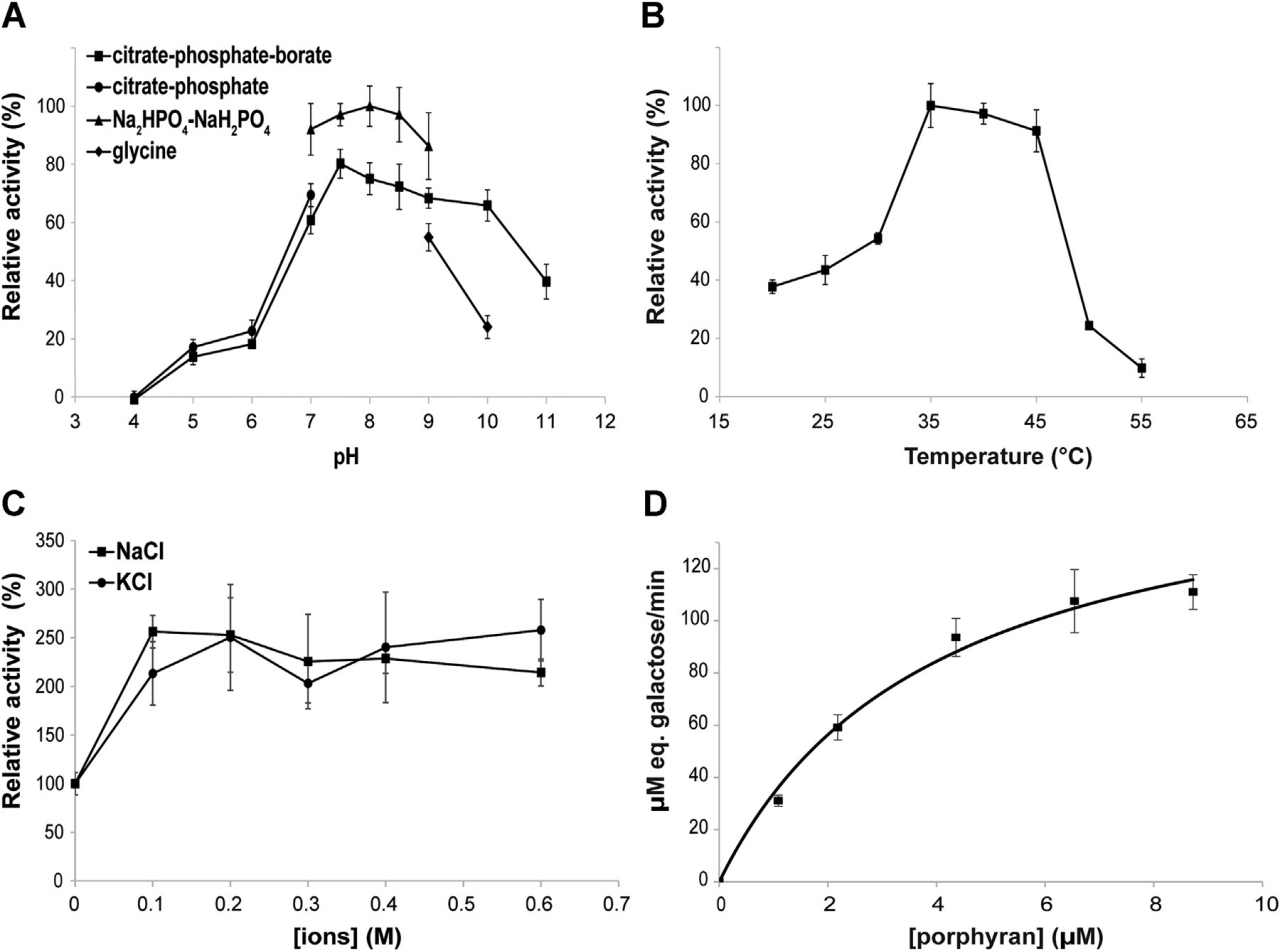
**Biochemical characteristics of CcGH16-3 performed with an enzyme concentration between 35.2 nM and 356.4 nM.***A*, enzyme activity at different pHs, (*B*) enzyme activity at different temperatures, (*C*) influence of NaCl and KCl concentrations, (*D*) kinetic curve with 50 mM Na_2_HPO_4_/NaH_2_PO_4_ buffer pH 8.0 in the presence of 1 mM MgCl_2_ and 100 mM NaCl at 35 °C and 35.2 nM CcGH16-3. All error bars refer to SD.

To determine the physicochemical properties of CcGH16-3, different enzymatic conditions were tested *in vitro* using reducing sugar assays and 0.2% porphyran at room temperature (RT). The CcGH16-3 protein showed optimal activity between pH 7.5 and 8.0 (Fig. 5*A*) but with a fairly stable activity in a large pH range (pH 7.0–9.0). Other bacterial GH16 β-porphyranases, WfPor16A and WfPor16C, have been characterized as having pH optimum at pH 6 and 7, respectively, also with fairly stable activity over a broad pH range (44, 55). CcGH16-3 retained more activity at pH 10.0 in phosphate buffer compared to glycine buffer. CcGH16-3 activity was significantly reduced in Tris buffer (not shown) relative to the phosphate buffer. Phosphate might stabilize the protein activity (44, 55) and/or Tris and glycine may inhibit the CcGH16-3 enzymatic activity. CcGH16-3 prefers a temperature range between 35 °C and 45 °C with a rapid increase in activity between 30 °C and 35 °C and a rapid decrease between 45 °C and 50 °C (Fig. 5*B*). The optimum temperature of 35 °C to 40 °C is similar with that of WfPor16A (40 °C) and WfPor16C (35 °C) (44, 55).

NaCl and KCl as well as divalent cations (Mg^2+^ and Ca^2+^) have been shown to play a role in the activity of β-porphyranases (44, 55). To observe the effect of salts on the activity of CcGH16-3, different concentrations of NaCl and KCl were tested and an increase in activity was observed for both 0.1 M NaCl and 0.1 M KCl (Fig. 5*C*). Addition of 1 mM MgCl_2_ and CaCl_2_ resulted in a 1.5-fold increase in enzyme activity, and Mn^2+^ and Cu^2+^ ions inhibited the activity with complete inhibition for the Cu^2+^ ion. Surprisingly, MgSO_4_ caused a 33% decrease in activity, suggesting that the sulfate ion inhibits activity (Table 1). Sulfate inhibition might be related to an enzyme interaction with a sulfate molecule within the active site cleft.

**Table 1:** Effect of metal ions on the activity of CcGH16-3

Ions (1 mM)	Relative activity (%)
w/o	100.0 ± 11.6
MgCl_2_	147.7 ± 15.4
MgSO_4_	67.3 ± 10.6
CaCl_2_	154.5 ± 1.9
MnCl_2_	70.7 ± 14.5
CuCl_2_	−5.6 ± 2.3

The porphyranase activity was measured in the following reaction conditions: 0.2% porphyran at 25 °C in 5 mM Tris–HCl pH 8.0, 10 mM NaCl with or without (w/o) 1 mM metal ions. Enzyme concentration used was 89.1 nM for MgCl_2_ and CaCl_2_, 178.2 nM for MgSO_4_ and MnCl_2_, and 356.4 nM for CuCl_2_.

To help determine the kinetic parameters of the CcGH16 enzyme for its porphyran polysaccharide substrate, we analyzed the porphyran extracted from *P. dioica* using multiangle light scattering (MALS) and ascertained its average molecular weight (MW) to be 458.6 ± 3.7 kg mol^−1^ (Fig. S3). A steady state Michaelis–Menten kinetic analysis was done using a reducing sugar assay in the presence of different concentrations of porphyran (1.09–8.72 μM) with 50 mM Na_2_HPO_4_/NaH_2_PO_4_ buffer pH 8.0 in the presence of 1 mM MgCl_2_, 100 mM NaCl, and 35.2 nM CcGH16-3 at 35 °C (Fig. 5*D*). CcGH16-3 demonstrated a *K*_*M*_ of 4.0 ± 0.8 μM, a *k*_cat_ of 79.9 ± 6.9 s^−1^, and a *k*_cat_/*K*_*M*_ of 20.1 ± 1.7 μM^−1^ s^−1^. The CcGH16-3 *K*_*M*_ is similar to the enzymes WfPor16A (3.16 μM) (55) and WfPor16C (3.22 μM) (44) from the marine bacterium *Wenyingzhuangia fucanilytica*; however, the CcGH16-3 *k*_cat_ is higher than WfPor16A (13.41 s^−1^) (55) and WfPor16C (50.14 s^−1^) (44). The CcGH16-3 has an improved *k*_cat_/*K*_*M*_ as well (20.1 ± 1.7 μM^−1^.s^−1^ relative to 4.25 μM^−1^ s^−1^ and 15.57 μM^−1^ s^−1^ for WfPor16A (55) and WfPor16C (44), respectively).

### Identification of *CcGH16-3* active site

The GH16 family uses a double displacement mechanism (56) for hydrolysis, which passes through an enzyme-glycosyl intermediate and results in stereochemical retention of configuration at the anomeric carbon. As briefly discussed in the introduction, two different types of active sites have been characterized in the GH16 family: the ExDxE motif, which includes lichenases and β-glucanases (57, 58), and the β-bulge ExDxxE motif, which includes bacterial carrageenases, agarases, and porphyranases (39, 54, 59). An amino acid sequence alignment of the annotated *C. crispus* GH16 enzymes with known bacterial GH16 β-porphyranases (36, 44, 55) as well as with the two closest homologs from red algae (26, 60) was performed. Unsurprisingly, the sequence alignment shows that the CcGH16-3 active site belongs to the ExDxxE motif with red seaweed polysaccharide specificities (Fig. S1) (34, 36, 37).

**Figure 6:**
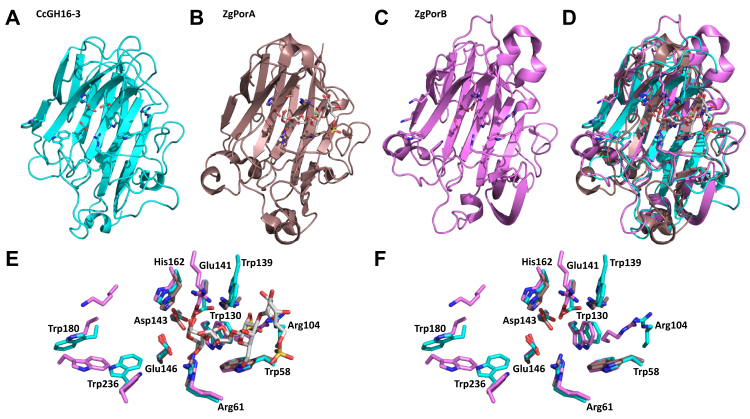
**Structural comparison of CcGH16-3 (CcPorA), ZgPorA and ZgPorB.***A*, AlphaFold structure of CcGH16-3 in *cyan*. *B*, crystal structure of ZgPorA (pdb id 3ilf) active site mutant in *dirty violet* in complex with oligoporphyran DP4. *C*, crystal structure of ZgPorB (pdb id 3juu) in *pink*. *D*, secondary structure overlap of all the structures, same color scheme as above. *E*, overlap of the active site amino acids suggesting possible subsites including oligoporphyran DP4 from the ZgPorA complex, only CcGH16-3 amino acids are labeled for clarity. *F*, overlap of the active site amino acids suggesting possible subsites, again only CcGH16-3 amino acids are labeled for clarity.

To investigate subtleties in active site substructure of CcGH16-3, a structural comparison was performed between CcGH16-3, ZgPorA, and ZgPorB (Fig. 6). The UniProt (61) AlphaFold (62) model of CcGH16-3 (R7Q792_CHOCR) demonstrates a long rather open active site with the predicted ExDxxE residues (E141, D143, and E146) within the cleft oriented towards the solvent (Fig. 6, *A*, *E* and *F*). This open cleft suggests longer chain endolytic nonprocessive hydrolase activity. There are five tryptophan residues (W58, W130, W139, W180, and W236) exposed to the solvent along the open active site cleft, which are potential hydrophobic platforms for interaction with porphyran and suggest potential subsites. There are also two arginine residues (R61 and R104), which may provide important salt bridging interactions with sulfate residues along the porphyran polymer chain. Several of the tryptophans (W58, W130, and W236), H162, and both arginines are structurally conserved with ZgPorB (Fig. 6, *C*–*F*). There is much less overlap with ZgPorA within its more closed active site, though W58, R61, and H162 are structurally conserved (Fig. 6, *B* and *D*–*F*).

**Figure 7:**
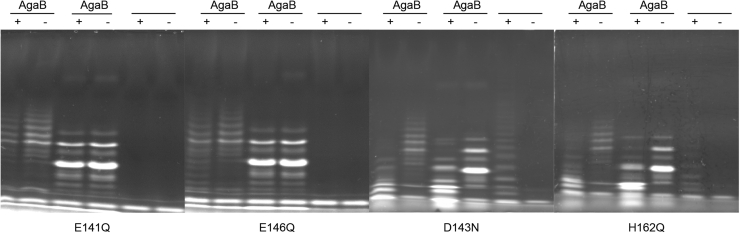
**FACE of CcGH16-3 active site mutant enzyme reaction products after an overnight digestion.** CcGH16-3_E141Q and CcGH16-3_E146Q are inactive on oligoporphyran produced by ZgAgaB and porphyran (34). CcGH16-3_D143N and CcGH16-3_H162Q are active on oligoporphyran produced by ZgAgaB and porphyran. The (+) symbol represents the enzymatic digestion by the CcGH16-3 mutant listed below each gel. The negative control (−) was the glycans with buffer A (10 mM Tris–HCl pH 8.0 and 100 mM NaCl). High MW oligoporphyran substrates (F21-F22) produced by ZgAgaB are in lanes 1 and 2, low MW oligoporphyran substrates produced by ZgAgaB (F5-F16) are in lanes 3 and 4, and porphyran polysaccharide substrate is in lanes 5 and 6. Samples were labeled with the ANTS fluorophore and separated by PAGE. Concentration of enzyme used was 0.28 μM for CcGH16-3, 0.58 μM for E141Q, 2.67 μM for E146Q, 1.99 μM for D143N, and 0.41 μM for H162Q. FACE, fluorophore-assisted carbohydrate electrophoresis; MW, molecular weight.

To confirm their contribution to the enzyme active site, four conserved residues were selected for mutagenesis studies: the two catalytic glutamates, E141 and E146, having nucleophile and acid/base function, respectively, D143, which is hypothesized to accelerate deglycosylation of the enzyme-glycosyl intermediate, and H162, which may be involved in proton transfer with D143 and thus also involved in the deglycosylation step (63, 64) (Fig. S1). The mutants CcGH16-3-E141Q, CcGH16-3-D143N, CcGH16-3-E146Q, and CcGH16-3-H162Q were tested *in vitro* on porphyran and with ZgAgaB treated porphyran. The mutant enzyme concentrations for E141Q, E146Q, D143N, and H162Q were 2.1×, 9.5×, 7.1×, and 1.5× higher, respectively, than the CcGH16-3 concentration. No degradation was observed by FACE for the two substrates with the mutant E141Q and E146Q (Fig. 7) and this was confirmed by reducing sugar assay (data not shown). The mutants D143N and H162Q showed activity on both substrates as determined by FACE (Fig. 7); however, reducing sugar assays showed extremely low residual activity for only H162Q (data not shown). The reactions for FACE ran overnight and thus likely to completion, whereas the reactions for the reducing sugar assays ran for 5 min, which would explain the discrepancy between the results from the two assays. This residual activity of D143N correlates with previous catalytic analyses on the GH16 endo-β-1,3/β-1,4-glucanase enzyme from *Bacillus macerans*, and it is not unexpected that H162Q has a similar effect given the predicted role of both of these residues in proton transfer during the deglycosylation step (63, 64).

### Mass spectrometry structural characterization of digested porphyran products

Characterization of bacterial β-porphyranases has revealed complex specificities, reflecting the complexity of the polysaccharide substrate (44, 54, 65). In the following discussion, we use Knutsen’s nomenclature for red algal glycans (66); thus, D-galactose is represented by a G, L-galactose-6-sulfate by an L6S and 3,6-anhydro-L-galactose by an LA residue. Me represents a methyl group.

To investigate the specificity of CcGH16-3, mass spectrometry (MS) analyses were performed. Electrospray ionization (ESI)-MS spectra were acquired to obtain a complete mass profile for (1) purified low MW oligoporphyrans (fractions F21-F22), (2) purified high MW oligoporphyrans (fractions F5-F16), both low and high MW fractions were produced by pretreating porphyran with ZgAgaB, and (3) porphyran polysaccharide. The products of the reaction were also analyzed after incubation with CcGH16-3. Hexylammonium (adducts, C_6_H_16_N^+^ + 102.127 Da) was used as classical ion pairing reagent to prevent the “in-source desulfation” of the oligosaccharides (67).

### ESI-MS determined porphyranase activity

**Figure 8:**
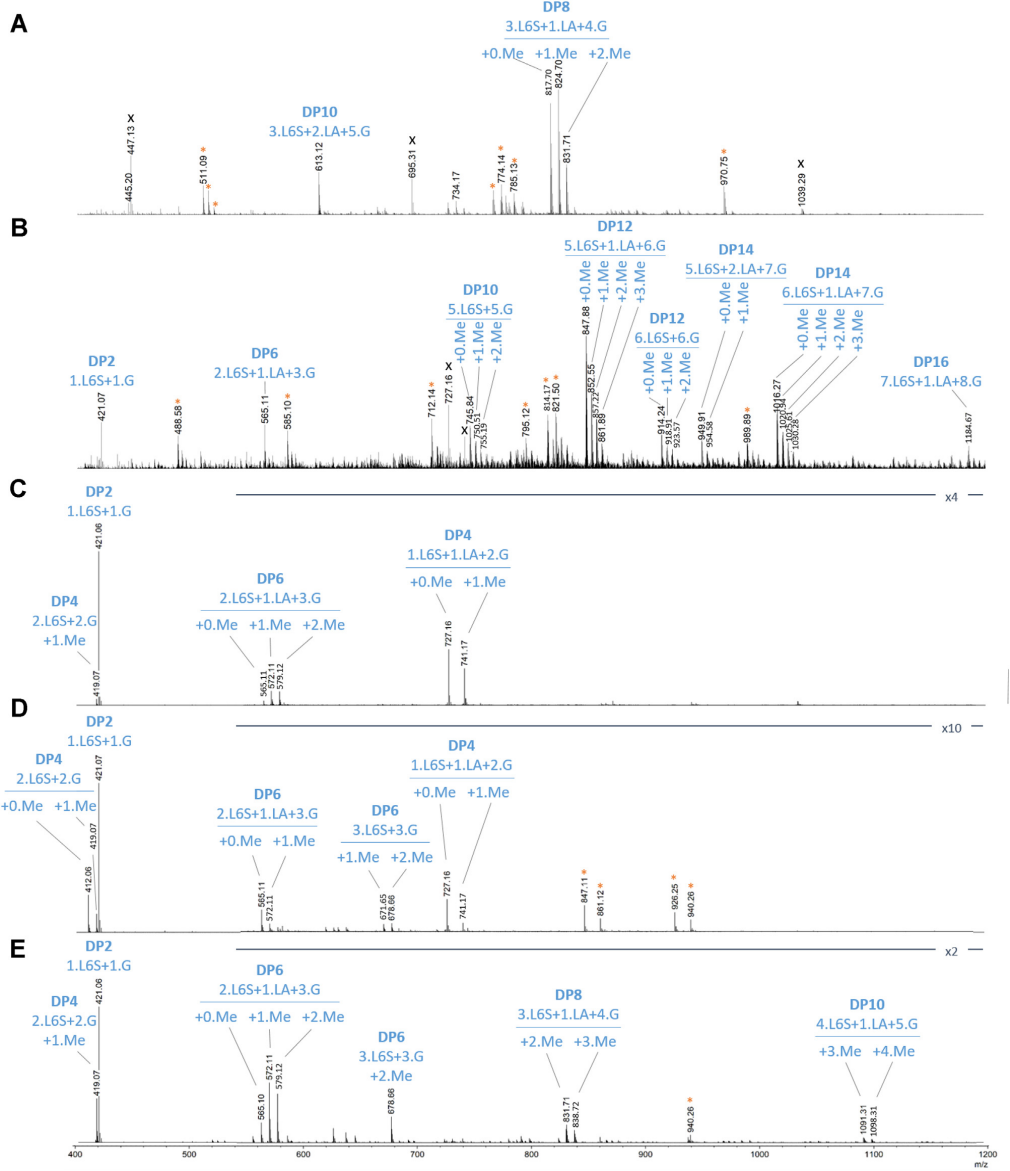
**ESI-MS(−) spectra.***A*, low MW oligoporphyrans (fractions F21-F22) and (*B*) high MW oligoporphyrans (fractions F5-F16), both produced by pretreating porphyran with ZgAgaB. The three CcGH16-3 digestions on (*C*) low MW oligoporphyrans (fractions F21-F22), (*D*) high MW oligoporphyrans (fractions F5-F16), both produced by pretreating porphyran with ZgAgaB, and (*E*) porphyran polysaccharide. Annotations were deduced from the exact mass measurements. *Orange stars* indicate minor adducts or charge states from an annotated species. X indicates a contaminant. The structures were deduced from the exact mass measurements. ESI-MS, electrospray ionization-mass spectrometry; MW, molecular weight.

From the ESI-MS analyses (Fig. 8) and in agreement with the FACE analysis (Fig. 4), a porphyranase activity was confirmed. On the product spectra of the three samples after incubation with CcGH16-3 (Fig. 8, *C*–*E*), pure oligoporphyrans were identified as the major products: DP2 (1.L6S+1.G as singly charged species at m/z 421.0) and DP4 (2.L6SG+2.G as doubly charged species with 0 Me at m/z 412.0 for high MW oligoporphyrans and 1 Me at m/z 419.0 in the three samples). Additionally, pure DP6 was identified in the CcGH16-3 digested high MW oligoporphyrans (3.L6SG+3.G with 1 Me at m/z 671.6 and 2 Me at m/z 678.6) and in the porphyran polysaccharide extract (3.L6SG+3.G with 2 Me at m/z 678.6). These pure oligoporphyrans are missing in the digestion using only ZgAgaB (Fig. 8, *A* and *B*), with the exception of a minor DP2 in the high MW oligoporphyrans. To summarize these results, pure oligoporphyran DP2 and DP4 were the major products, in the oligosaccharide samples incubated with CcGH16-3. No pure agarose oligosaccharides were present, which further supports the CcGH16-3 porphyranase specificity, though mixed species containing both porphyranobiose and agarobiose were identified. Interestingly, in each sample, different specific species were produced allowing us to explore in more details the tolerances of the different catalytic subsites.

### Exploring the −1/+1 active site

To further explore the fine specificity of CcGH16-3, 18 oligosaccharide species were structurally characterized using a high-energy activation tandem MS approach (helium charge transfer dissociation, He-CTD) coupled with ultra-HPLC (UHPLC) in one or more reaction media (UHPLC-CTD-MS). With the aim to specifically separate isomers in the enzymatic milieu, we moved to heptylammonium, as ion pairing reagent, which is more classically used for the separation of uronic oligosaccharides with a higher p*K*a (68) (adducts, C_7_H_18_N^+^ + 116.143 Da). This allowed enhancement of the retention time of the low sulfated species and thus better separation efficiency on the C18 column.

In the low MW oligoporphyrans produced by ZgAgaB only (Fig. 8*A*), the ESI-MS analysis showed that three of the major products are DP8 (3.L6SG+1.LA+4.G with 0 Me (m/z 817.7), 1 Me (m/z 824.7), and 2 Me (m/z 831.7). The UHPLC-CTD-MS analysis revealed, for the monomethylated species, two isomers (respectively Fig. S4, *A* and *B*). The structural characterizations of each isomer revealed the isomerism is due to the positioning of the Me group (L6S-G-L6S-G-L6S-G(Me)-LA-G) (RT 14.7 min, major species) and L6S-G-L6S-G-L6S-G-LA-G(Me) (RT 15.1 min, minor species) (Table 2). This advances our understanding of ZgAgaB specificity as it is able to accommodate both methylated and unmethylated G (underlined) in its −1 active site subsite.

**Table 2:** Oligosaccharides structurally characterized by UHPLC-CTD-MS for the low MW oligoporphyran fraction F21-22 produced by ZgAgaB only and for the three samples digested by CcGH16-3: porphyran, F5-16 high MW ZgAgaB oligosaccharides, and F21-22 low MW ZgAgaB oligosaccharides

Blue circle: D-galactose, red circle: 3,6-anhydro-L-galactose, yellow circle: L-galactose-6S, black circle: 6-methyl-D-galactose. Major indicates an isomer is more abundant than a relatively minor abundant isomer.

All the UHPLC-CTD-MS structurally characterized oligosaccharide products of CcGH16-3 have strictly L6S on their nonreducing terminus (Table 2 and Figs. S5–S11). Therefore, the +1 subsite of CcGH16-3 accommodates L6S (Table 2). We structurally characterized several methylated oligosaccharide products from the CcGH16-3 digestion of porphyran using UHPLC-CTD-MS; none were identified with a reducing end methylated G (Table 2), supporting a specificity for unmethylated G in the −1 subsite and an intolerance to methylated G (Table 2). Complicating the aforementioned assessment is the presence of the DP4 1.L6S+1.LA+2.G+1Me that was characterized by UHPLC-CTD-MS only in the low and high MW oligosaccharide CcGH16-3 digestions to have the structures L6S-G(Me)-LA-G (majority) and L6S-G-LA-G(Me) (minority)–placing the methylated G potentially in the −1 subsite of CcGH16-3 (underlined). The most plausible explanation is that L6S-G-LA-G(Me) is already situated on the reducing end of the longer DP8 oligosaccharides produced by ZgAgaB before treatment with CcGH16-3 (Table 2, Figs. S4 and S12). Overall, these results indicate the subsites −1 and +1 of CcGH16-3 are specific for unmethylated G and L6S, respectively.

### Length of the catalytic site

There was pure oligoporphyran DP4 detected in the high MW oligoporphyran CcGH16-3 digest (Fig. 8*C*); however, treatment by CcGH16-3 of the low MW oligoporphyrans and the porphyran polysaccharide resulted in only methylated DP4 2.L6S+2.G (Fig. 8*B*). This suggests that CcGH16-3 prefers oligosaccharides longer than DP4, though it is likely capable of cleaving the pure oligoporphyran DP4 2.L6S+2.G when pushed to its limit. This is supported by the long open active site predicted in the AlphaFold model (Fig. 6). The CcGH16-3 digest on porphyran (Table 2) produced the UHPLC-CTD-MS characterized DP6 products L6S-G-L6S-G-LA-G and L6S-G-L6S-G(Me)-LA-G, which each have a potential nonreducing end CcGH16-3 cleavage site after their G residues (underlined) uncut by the enzyme. This suggests that there are more than two negative subsites in CcGH16-3. Based on the AlphaFold model, the catalytic acid/base residue sits in the expected −1 subsite, W236 might provide the −2 subsite and W180 may provide the −3 subsite (Fig. 6, *E* and *F*). Similarly, from the porphyran CcGH16-3 digest (Table 2), the DP6 oligosaccharide products L6S-G-LA-G-L6S-G and L6S-G(Me)-LA-G-L6S-G have a potential CcGH16-3 cleavage site after their G residues (underlined) closer to the reducing end that are uncut by the enzyme. This suggests there are more than two positive subsites for CcGH16-3. Based mainly on the structural overlap of CcGH16-3 with ZgPorA in complex with oligoporphyran DP4, W130 and the catalytic nucleophile E141 are situated to be the +1 subsite, W58 and R104 the +2 subsite, and W139 potentially participating as the +3 subsites (Fig. 6, *E* and *F*). Finally, the active site subsites of the bacterial homologs ZgPorA and ZgPorB have been characterized between −3 and +3; therefore, we have undertaken a similar subsite dissection for CcGH16-3 (36).

### Fine catalytic specificity through subsite dissection

**Figure 9:**
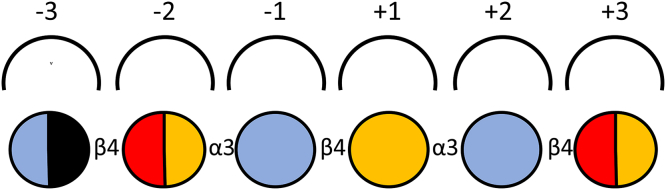
**Schematic representation of the subsite-binding specificity of the catalytic cleft of CcGH16-3 from *C. crispus*.** Subsites are represented by semicircular lines above the scheme of a bound sugar chain and numbered according to convention (87). The different symbols used for sugar units are *blue*: D-galactose, *red*: 3,6-anhydro-L-galactose, *yellow*: L-galactose-6S, *black*: 6-methyl-D-galactose, *white*: positions are not necessarily occupied for cleavage to occur. The presence of a multicolored circle indicates that various monosaccharide units can be accommodated by the subsite.

The CcGH16-3 digested porphyran characterization by UHPLC-CTD-MS identified structural isomers in fine detail from DP6 to DP8 (Table 2). The −2 subsite of CcGH16-3 can accommodate LA or L6S as revealed by the hexasaccharide products L6S-G(Me)-L6S-G-LA-G and L6S-G-LA-G-L6S-G (an LA is found in position −2 in 9 of 16 CcGH16-3 product structures). The −3 subsite can accommodate G or G(Me) as demonstrated with the product structures L6S-G-LA-G-L6S-G and L6S-G-LA-G(Me)-L6S-G (a methylated G in −3 is observed in 9 of the 16 structures). The L6S-G-LA-G-L6S-G and L6S-G(Me)-LA-G-L6S-G structures allow us to deduce that the +2 subsite shows a certain flexibility and can accommodate G with or without methylation on the carbon 6 hydroxyl group (a methylated G in +2 is observed in 8 of 16 product structures). The +3 can accommodate either LA or L6S as revealed by the hexasaccharides L6S-G-LA-G-L6S-G and L6S-G(Me)-L6S-G-LA-G (an LA is found in +3 in 7 of the 16 product structures). This has led us to postulate a schematic representation of the subsite-binding specificity of the catalytic cleft for CcGH16-3 that is shown in Figure 9 and is formatted for comparison to the schematic proposed by Hehemann *et al.* (36).

Comparison to ZgPorA and ZgPorB reveal the CcGH16-3 cleavage site has more substrate-binding plasticity than the ZgPorA and ZgPorB porphyranases. ZgPorA can only accommodate L6S in the −2 subsite and G in the +2 subsite. ZgPorB can only accommodate G in the +2 subsite (36). CcGH16-3 also differs from WfPor16C, which can recognize a methylated G at its −1 subsite (44). Overall, the CcGH16-3 specificity most closely resembles that of the porphyranase WfPor16A, at least between −2 to +2 subsites (55). Structurally, these differences in substrate binding for CcGH16-3 may be at least partially mediated by its open concave active site groove and the presence and orientation of the basic amino acids and tryptophan residues lining the groove (Fig. 6, *A*, *E* and *F*).

## Conclusion

The discovery of porphyranase activity in a GH16 enzyme produced by the carrageenophyte *C. crispus* was surprising and promptly invalidated the hypothesis that the CcGH16-3 enzyme is involved in carrageenan metabolism. Since porphyran and porphyranobiose motifs are not known components of the *Chondrus cripus* ECM, what biological role does CcGH16-3, a porphyranase, have in *C. crispus*?

CcGH16-3 could potentially act on minority porphyran motifs not yet identified in *C. crispus*. Alternatively, it could have a role in defense against a parasitic red alga with an ECM containing porphyran (69). Red algal parasitism is common in the Florideophyceae (70), which includes *C. crispus* as a member. There are over 100 known red algal parasitic species in over 60 genera and a host defense against these evolutionarily related pathogens, which often infect close relatives, could provide a distinct evolutionary advantage (71). The genetic similarity between red algal host and parasite may be a key strategy in host evasion (71). Red algal parasites fuse with host cells to form secondary pit connections; a glycoprotein pit plug seals the cell to cell connection (70). Any successful red algal cell parasitic fusion event would thus require ECM modification. Recognition and protection against closely related red algal parasites though attack on structurally distinct glycan structures in the ECM could defend against fusion with the host cell and provide an important enzymatic host defense against red algal parasites.

The red alga *Pyropia yezoensis* produces an alginate lyase (11) and the red alga *Chondrus verrucosus* produces three chitinases (72); however, neither alginate nor chitin is known to be part of their ECMs. A predicted biological role for these enzymes is their involvement in self-defense against pathogens such as bacteria, brown algae, fungi, and grazing marine animals. In plants and animals, pathogen attack can lead to release of molecules, including oligosaccharides (73), which act as elicitors of defense reactions (74). If these elicitors are from exogenous sources, they are considered Pathogen/Microbial Associated Molecular Patterns (P/MAMPs), which are recognized by plasma membrane pattern recognition receptors generating signaling cascades that activate host innate immune responses (2, 74). The innate immune response can include the production of ECM-active enzymes, which target the attacker (*e.g.*, lysozyme, chitinases, and glucanases) (2, 74). Thus, if agaran/porphyran oligosaccharides are released from nearby red algae by CcGH16-3, these oligosaccharides might act as exogenous elicitors of algal defense reactions (2).

Localization of CcGH16-3 in the Golgi or endoplasmic reticulum would support a role in the biosynthesis of the ECM or its involvement in the maturation of glycoproteins. Or, if CcGH16-3 is released into the external environment, it could contribute to positive fitness in niche colonization and light competition by discouraging the colonization nearby by porphyranophyte/agarophyte red algal competitors. Predictions using eukaryotic protein localization programs such as used in (75) suggest that CcGH16-3 is cycled into the secretory pathway of the organism, though the exact localization remains unclear and histochemical microscopy experiments were inconclusive at best.

Our findings open the spectrum of biochemical activities found in red algae that are associated to polysaccharides unknown to be found in the host ECM. Precautions have to be made with *in silico* annotations of algal carbohydrate-active enzymes and biochemical studies are essential to confirm substrate specificity. Moreover, a major biological question remains open about the physiological function of a transversal specificity that requires elucidation of enzyme localization, fine ECM examination for minor glycan motifs, and studies in complex environmental conditions.

## Experimental procedures

### cDNA preparation

Extraction of mRNA from *C. crispus* cells was done as previously described (19). The cDNA preparation was done with the GoScript Reverse Transcription System (Promega) according to manufacturer’s instructions.

### Phylogenetic analysis

The GH16 members were extracted from the CAZy database (July 2021) (23) and used to retrieve amino acid sequences from GenBank. Based on the taxonomic diversity and giving preference to those that were biochemically characterized, five sequences were selected for each of the GH16 subfamilies that have closest similarity to the algal enzyme sequences, which are GH16_11 through GH16_17 and GH16_26 (Supporting Information File 1). Five representative sequences from GH16_3 (which are laminarinases) were included as an outgroup. The sequences were analyzed to reveal the modular structure using BLAST (76) and Multalin (77) and only the catalytic modules were kept for further steps. The sequences of the catalytic modules were aligned with MUSCLE (78) using the G-INS-i (Iterative refinement, using WSP and consistency scores, of pairwise Needleman-Wunsch global alignments) strategy. A maximum-likelihood phylogenetic tree was estimated with RAxML (79) (100 bootstrap replicates) and visualized with iTOL (80).

### Intergenic amplification of *C. crispus* genomic DNA

An intergenic DNA fragment was amplified by PCR with the primers *chc_t00002034001*-F and *genccgh16-3*-R (Table S2) using tetrasporophyte genomic DNA as a template and GoTaq Flexi DNA Polymerase (Promega). The gene fragment was extracted from the agarose gel then sequenced using 3130xl Genetic Analyzer (Applied Biosystems) with the same primers used for PCR.

### Algae, bacterial strains, and plasmids

*C. crispus* was collected in Roscoff on August 16, 2018, as previously described (19). *P. dioica* was collected in August 5, 2015, at Sibiril (GPS coordinates 48.691801341672004 −4.085713118736136). *E. coli* stellar (Takara) and Bl21(DE3) were used as plasmid hosts and for protein overproduction, respectively (Table S3). The plasmid pRF3 is a derivative of pFO4 with one less *NheI* site (46). First, 1090 bp from pFO4 using *PstI* and *EcoRI* were ligated to a pGEX-4T1 *PstI*/*EcoRI* fragment, leading to the intermediate vector pRF1. Then, the plasmid pRF1 was linearized with *NheI* and the sticky ends were removed using Mung Bean Nuclease (NEB) before recircularization by T4 DNA ligase (NEB) (pRF2). At the end, the 1086 bp *EcoRI*/*PstI* fragment of pRF2 was reinserted in the 4617 bp *EcoRI*/*PstI* pFO4 fragment that forms pRF3.

### Cloning and site-directed mutagenesis

The *ccgh16-3* gene was amplified from *C. crispus* cDNA by PCR using the Phusion high-fidelity DNA polymerase (NEB) with the primers *ccgh16-3*-F/R (Table S2). The PCR product and the pRF3 plasmid were digested by *BamHI* and *EcoRI*, which were then ligated using T4 DNA ligase (NEB). The resulting recombinant protein has an N-terminal hexahistadine-tag. Four different active-site mutants were also constructed using site-directed mutagenesis. Four primer couples targeted the E141 to Q (mutant E141Q), D143 to N (mutant D143N), E146 to Q (mutant E146Q), and H162 to Q (H162Q) (Table S2). The QuikChange II site-directed mutagenesis kit (Stratagene) was used to obtain the mutants by PCR from the pRF3_*ccgh16-3*_ plasmid template.

### Heterologous protein production of CcGH16-3 and mutants

The plasmids were transformed into the *E. coli* BL21(DE3) expression strain. A preculture in Luria-Bertani medium was incubated at 37 °C overnight and then diluted at 1:100 in fresh 1 l terrific broth medium for cell growth at 37 °C until reaching an absorbance at 600 nm of ∼2 to 2.5. The protein production was induced with 1 mM IPTG at 20 °C overnight. The culture was centrifuged 30 min, 7656*g* at 4 °C and the pellet was chemically lysed. The pellet was resuspended with 15 ml of resuspension buffer (50 mM Tris–HCl, pH 8.0, 25% sucrose, and 10 mg lysozyme) and stirred for 15 min. The resuspended cells were then lysed with 30 ml of lysis buffer (1% sodium deoxycholate, 1% Triton X-100, 20 mM Tris–HCl, pH 7.5, and 100 mM NaCl) and stirred 5 min at 4 °C. A volume of 200 μl of 1 mg/ml DNAse I was added and MgCl_2_ to a final concentration of 5 mM to the lysate. The lysate was then incubated at RT until the viscosity decreased and then centrifuged 30 min, 26,915*g* at 4 °C. The soluble fraction was filtered at 0.2 μm before purification using the AKTA purifier (Amersham Biosciences). A HisTrap NTA column with cobalt (GE Healthcare) was equilibrated with buffer A (50 mM Tris pH 8.0, 100 mM NaCl, and 10 mM Imidazole) and the elution was done with a linear gradient of imidazole by mixing buffer A and buffer B (50 mM Tris pH 8.0, 100 mM NaCl, and 500 mM Imidazole) up to 100% buffer B. The peak fractions were concentrated with an Amicon 10 kDa cutoff (Merck) until the desired volume to continue with size-exclusion chromatography (SEC) on a Sephadex S200 column (GE Healthcare) with buffer C (50 mM Tris pH 8, 100 mM NaCl). The protein concentration was measured by nanodrop using an extinction coefficient of 77,585 l mol^−1^ cm^−1^ as calculated using the ProtParam - ExPASy on-line tool (81). The yields of recombinant CcGH16-3 and the mutant enzymes were ∼6 mg per liter of culture.

### Marine polysaccharide and oligosaccharide substrates

Carrageenan oligosaccharides were made in house using previously described methods (49). Porphyran was extracted from *P. dioica*. Briefly, the dried algae (50 g) was crushed with a blender and heated at 100 °C in 1 l ethanol 80% over 2 h. Once cool, the ethanol wash was discarded after sedimentation and a second ethanol wash of the sediment was performed. The sediment was mixed with 2 l NaCl 0.1 M and kept overnight at RT. The mix was warmed to 60 °C for 4 h, followed by centrifugation (45 min, 5000*g*, RT). The supernatant was transferred to 3 l 96% ethanol under strong stirring for polysaccharide precipitation. The precipitate was filtrated on a nylon membrane and dried overnight at 40 °C. Finally, the precipitate was dialyzed in water (MWCO 6–8000 Da) and lyophilized. Oligoporphyrans produced by *Zobellia* *gallactanivorans* ZgAgaB or ZgPorA digestion were produced as previously described (36, 51, 54).

### Porphyran MW by SEC-MALS

Average MW and size distribution of porphyran were measured by SEC-MALS using an Optilab rEX Refractive Index detector and a Dawn Heleos MALS detector (Wyatt Technology). The separation of the molecules was performed using a Thermo Ultimate 3000 chromatographic system and 3 Shodex SB-805-HQ, SB-804-HQ, and SB-803-HQ columns connected in series. Elution was performed in 0.1 M LiNO_3_ with 300 μg/l NaN_3_ at 0.5 ml/min. One hundred microliters (100 μl) of 0.1% (w/v) porphyran solution was injected on the system after filtration on a 0.45 μm membrane. Data were processed using the Astra software (Wyatt Technology), using a dn/dc ratio of 0.146 as suggested by the detector supplier.

### *In vitro* polysaccharide degradation assays

Porphyranase activity was measured with 0.2% porphyran at 25 °C in 5 mM Tris–HCl pH 8.0, 100 mM NaCl, and 0.28 μM CcGH16-3 as standard. The experiments with the mutants E141Q, E146Q, D143N, and H162Q were done at 0.58, 2.67, 1.99, and 0.41 μM, respectively. An *in vitro* reducing sugar method, adapted from Kidby and Davidson, was used (54). Briefly, 180 μl ferrycyanide solution (0.91 mM K_3_(CN)_6_, 273.6 mM Na_2_CO_3_, 5 mM NaOH) was mixed with 20 μl reaction. The mixture was incubated 15 min at 95 °C, then cooled down at RT. The absorbance was read at a wavelength of 420 nm by Spark (Tecan). The pH optimum was measured at 25 °C and in pH range of 4.0 to 11.0. Four different buffers were used with 50 mM citrate-phosphate buffer (Na_2_HPO_4_, pH 4.0–7.0), phosphate buffer (Na_2_HPO_4_/NaH_2_PO_4_, pH 7.0–9.0), citrate-phosphate-borate buffer (Na_2_HPO_4_, pH 4.0–11.0), and glycine buffer (pH 9.0–10.0). The temperature optimum was investigated in a range of 20 °C to 55 °C. All the ions were tested at 1 mM and NaCl/KCl in a range of 0.1 to 0.3 M. Another reducing sugar method with increased sensitivity, adapted from Lever (82), was used specifically for the kinetic curve. Briefly, 180 μl *p*-hydroxybenzoic acid hydrazide (PAHBAH) solution (0.5% PAHBAH_3_ in 0.5 M NaOH) was mixed with 20 μl reaction. The mixture was incubated for 5 min at 100 °C, then cooled down at RT. The absorbance was read at a wavelength of 410 nm by Spark (Tecan). The kinetic curve was obtained using different concentrations of porphyran (1.09–8.72 μM) with 50 mM phosphate buffer pH 8.0 in the presence of 1 mM MgCl_2,_ 100 mM NaCl, and 35.2 nM CcGH16-3 at 35 °C. Data were plotted by Origin program (Origin lab) and kinetic parameters were determined using nonlinear curve fitting according to the Michaelis–Menten equation.

### FACE

A volume equivalent to 40 μg of oligosaccharide or polysaccharide was used for FACE analysis (83). The enzymatic reactions were incubated at RT overnight and air dried in a speed vacuum. The pellet was resuspended with a volume of 2 μl of 0.15 M ANTS (8-aminonaphthalene-1,3,6-trisulfonic acid) in a solution of acetic acid and water (3:17) followed by 5 μl of 1 M sodium cyanoborohydride in dimethyl sulfoxide. The samples were incubated 37 °C overnight in the dark for labeling. Glycerol was added to a final concentration of 55% in each sample before deposing in the gel well. The gels were run for 2 h at 200 V in the dark at 4 °C on a 30% polyacrylamide gel. The gels were visualized under UV light.

### ESI-MS measurements

The mass measurements were performed on a SELECT SERIES Cyclic IMS (Waters) equipped with an ESI source. The instrument was operated in negative polarity in “V mode.” Samples were diluted in a solution of H_2_O/ACN/Hexylammonium acetate 20 mM pH 6 (1/2/1) and infused with a flow rate of 10 μl/min. Hexylamine was used to protect labile sulfate groups from ESI. A data table is provided in the Supporting Information (Table S4).

### LC-MS/MS analysis

The different samples containing the oligosaccharides were separated by ion-pair reversed-phase UHPLC using the system Acquity H-Class (Waters) coupled with a modified amaZon SL 3D ion trap mass spectrometer (Bruker Daltonics) for CTD measurements as introduced by Hoffman and Jackson (84).

The chromatographic separations were performed on an Hypersil GOLD C18 column (1 × 100 mm, pore diameter 175 Å and 1.9 μm porosity particles from ThermoScientific) heated at 45 °C. The flow rate was 0.175 ml/min. A ternary gradient was performed during 29 min (A: H2O, B: MeOH, C: 20 mM heptylammonium formate pH 5.9). Solvent B was ramped from 2% to 25% over 10 min, then increased to 73% at 23.5 min, and maintained for the next 4 min before moving to initial conditions. Solvent C was kept constant at 25% during all the gradient. Heptylamine was used as ion pairing agent to allow separation of the oligosaccharides and to prevent desulfation during ESI.

Mass measurements were performed using the following parameters: capillary voltage: 4.5 kV; nebulizer gas: 7.3 psi; dry gas: 4 l/min (80 °C). Mass spectra were acquired in positive ionization mode in the m/z range 350 to 2200. Activation of ions by CTD process was performed by interfacing the ion trap with a saddle field fast ion source (VSW/Atomtech) with helium as the CTD reagent gas. The helium flow from the ion gun was adjusted to get a vacuum of ∼1.2 × 10^−5^ mbar in the trap chamber. High voltage was applied using a high voltage generator (ION tech Ltd). Irradiation time was set to 200 ms with an energy around 6 keV.Raw data were converted thanks to the software MSConvert (http://proteowizard.sourceforge.net/tools.shtml) into mzML format to be used with mMass 5.5.0 (85) in order to annotate the different peaks with structures and fragments according to the nomenclature of Domon and Costello (86).

## Data availability

Data are contained within the main article and in the supporting information. For any queries regarding the article data contact the corresponding author.

## Supporting information

This article contains supporting information.

## Conflicts of interest

The authors declare that they have no conflicts of interest with the contents of this article.
