# Supplementary material for: Specificity of a β-porphyranase produced by the carrageenophyte red alga Chondrus crispus and implications of this unexpected activity on red algal biology
Source: J Biol Chem. 2022 Nov 17;298(12):102707. doi: 10.1016/j.jbc.2022.102707 (PMC9771727; doi:10.1016/j.jbc.2022.102707)
Supplement: Supplemental Figures S1–S12 and Tables S1–S4 [file mmc1.pdf]

Specificity of a β-porphyranase produced by the carrageenophyte red alga *Chondrus crispus* and implications of this unexpected activity on red algal biology

Manat G.[1], Fanuel M.[3,4], Jouanneau D.[2], Jam M.[1], Mac-Bear J.[3], Rogniaux H. [3,4], Mora T.[1], Larocque R.[2], Lipinska A.[1], Mirjam Czjzek[1], Ropartz D.[3,4], Ficko-Blean E.[1*]

[1]Sorbonne Université, CNRS, Integrative Biology of Marine Models (LBI2M), Station Biologique de Roscoff (SBR), 29680 Roscoff, Bretagne, France.
[2]Sorbonne Université, CNRS, FR 2424, Station Biologique de Roscoff, Roscoff, France.
[3]INRAE, UR BIA, F-44316 Nantes, France
[4]INRAE, BIBS facility, F-44316 Nantes, France

List of material included:

Figure S1
Figure S2
Figure S3
Figure S4
Figure S5
Figure S6
Figure S7
Figure S8
Figure S9
Figure S10
Figure S11
Figure S12

Table S1
Table S2
Table S3
Table S4

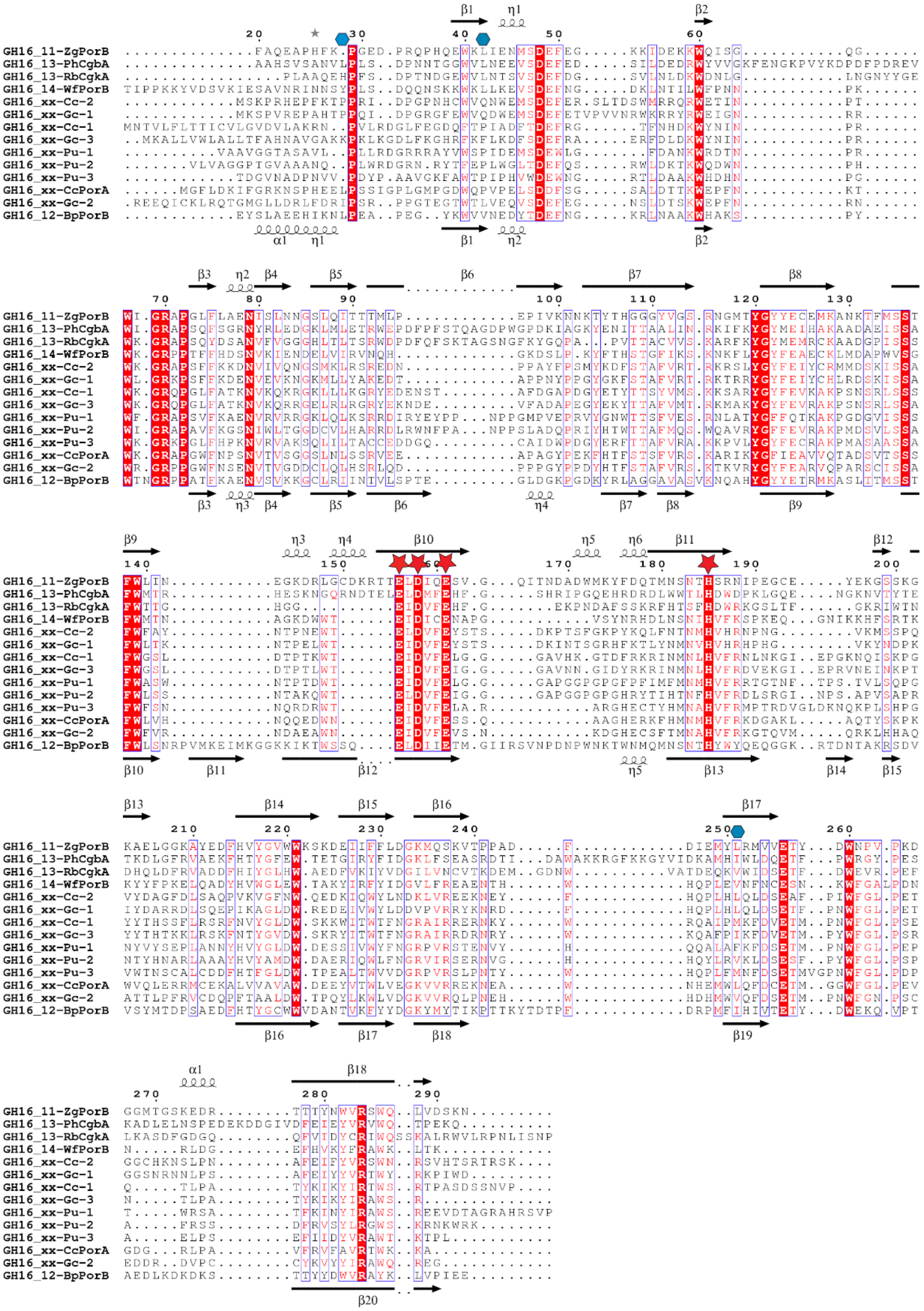

Figure S1. Multiple sequence alignment of the three CcGH16 and of selected GH16 porphyranases. All the amino acid sequences belong to biochemically characterized bacterial porphyranases except for *Gracilariopsis chorda* (Gc) and *Porphyra umbilicalis* (Pu) that are red algal GH16 members. The secondary structures from the structurally characterized ZgPorB (pdb id 3juu) and PbPorB (pdb id 4awd) are shown above and below the alignment, respectively. The red stars highlight the strictly conserved catalytic residues throughout GH16 enzymes. The blue hexagons demonstrate the amino acids that are different between the CcPorA/GH16-3 predicted from the genome sequence (shown) and the CcPorA/GH16-3 cloned from cDNA of *C. crispus* collected on the rocky shore. The alignment was done with MUSCLE and the figure was produced using ESPript version 3.0 software. Links to NCBI accession numbers and organism names can be found in Table S1.

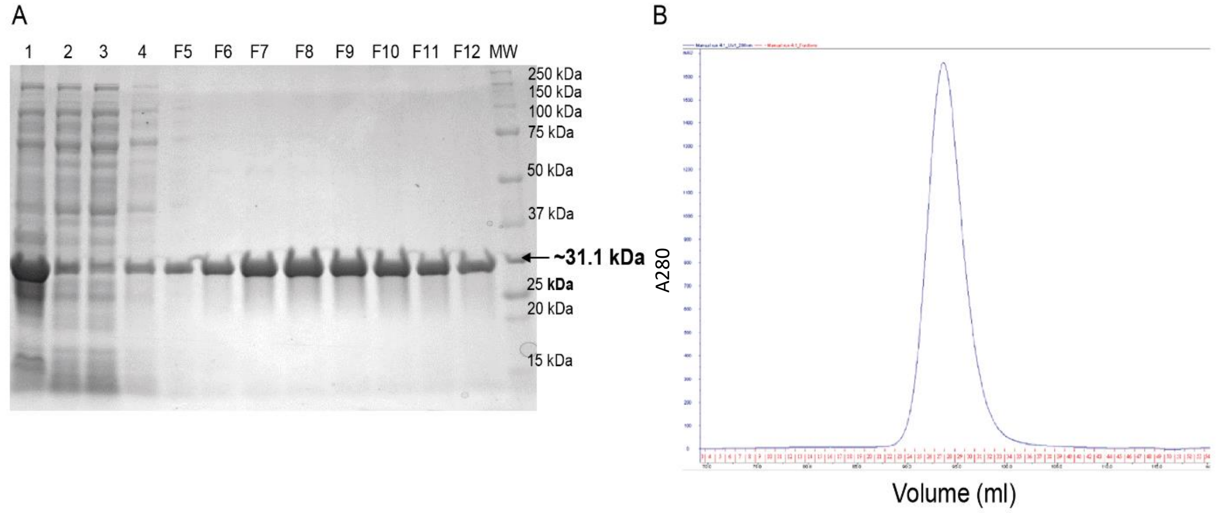

Figure S2. Gel electrophoresis of the CcGH16-3 production. CcGH16-3 is well produced in *E. coli* BL21(DE3). A. Purification using Co-NTA resin. Lane 1, insoluble fraction; lane 2, soluble fraction; lane 3, flow though; lane 4, 10 mM imidazole wash; F5-12, elution fractions (gradient 10-500 mM imidazole). B. Gel filtration profile shows only one peak at approximately 25 kDa confirming that the enzyme is monomeric.

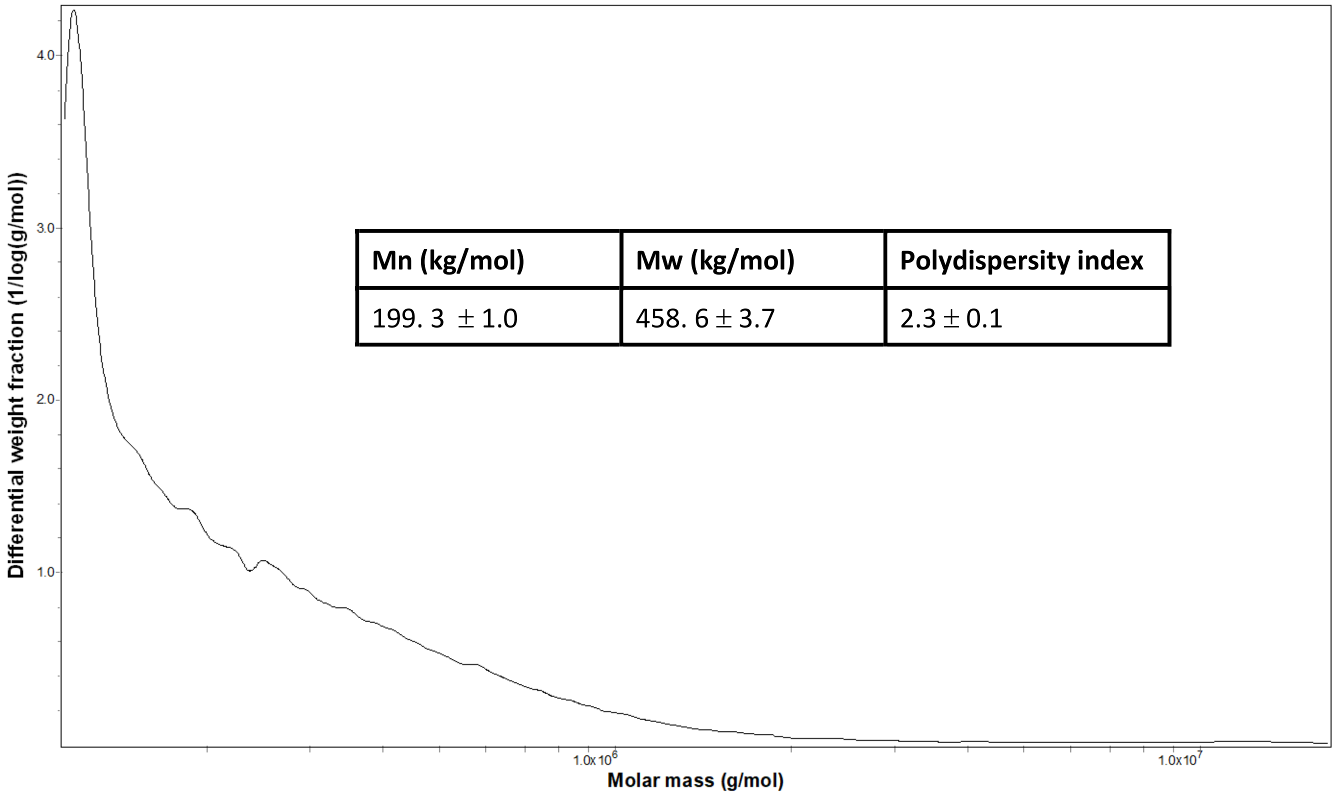

Figure S3. Molar mass distribution of porphyran polysaccharide extracted from *P. dioica* by MALS. Mn is number-average molar mass and Mw is weight-average molar mass. Polydispersity index is defined as Mw/Mn and reflects the heterogeneity of size distribution of the polymer.

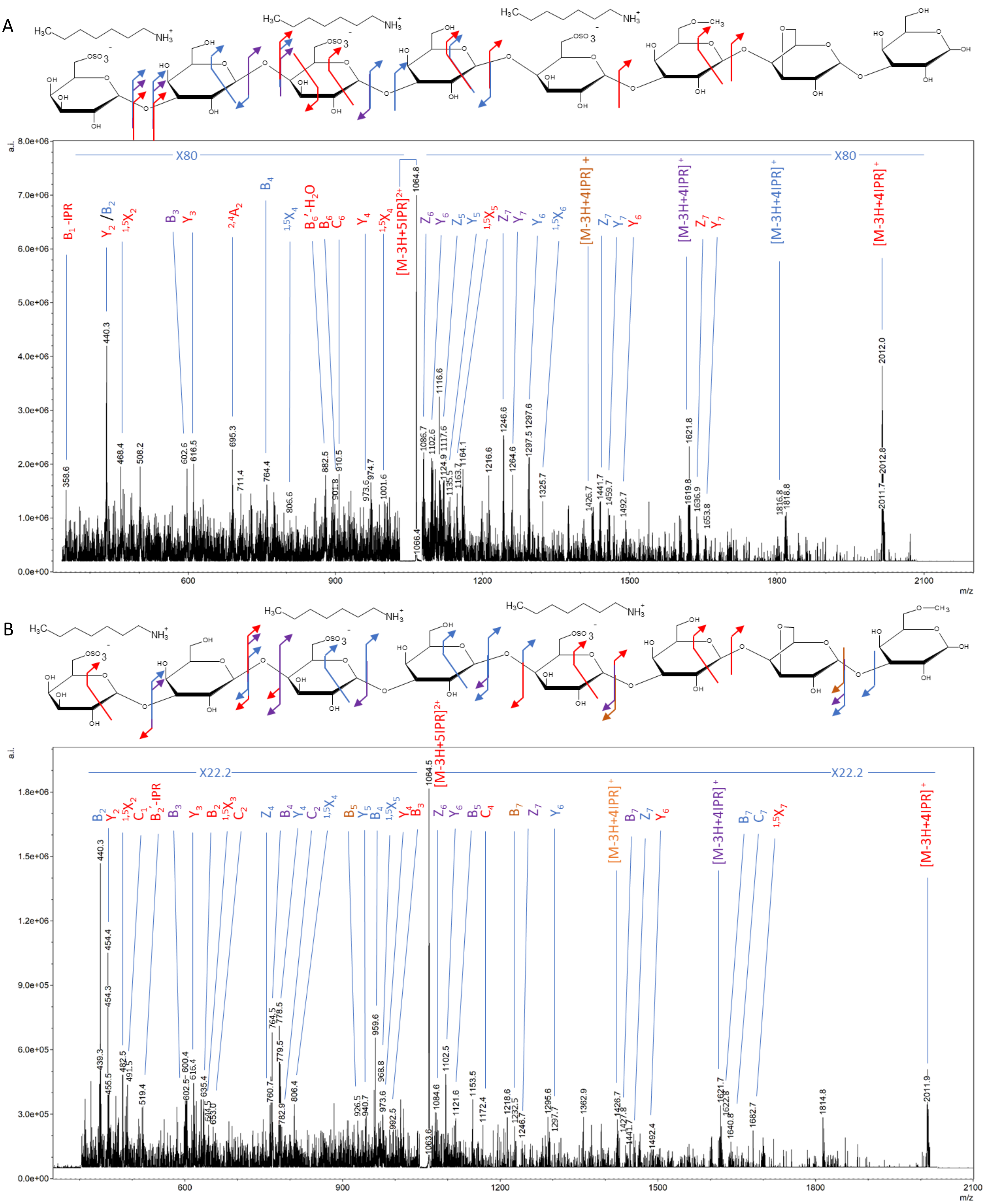

Figure S4. Helium charge transfer dissociation tandem MS spectra of $[M\text{-}3H+5IPR]^{2+}$ precursors at *m/z* 1064 (two isomers : A and B) in positive ion mode. The structures of the DP8 (3.L6S+1.LA+4.G+1.Me), L6S-G-L6S-G-L6S-G(Me)-LA-G and L6S-G-L6S-G-L6S-G-LA-G(Me), found in the sample with low MW oligo-porphyrans (F21-22) pretreated with ZgAgaB, are annotated with their fragments: red represents fully sulfated fragments; blue, fragments with one sulfate loss; purple, fragments with two sulfates losses and brown, three sulfates losses. A loss of the ion pairing reagent (heptylamine) is indicated as “-IPR” and one IPR (not represented on the schema) gives the charge state of the ion.

A

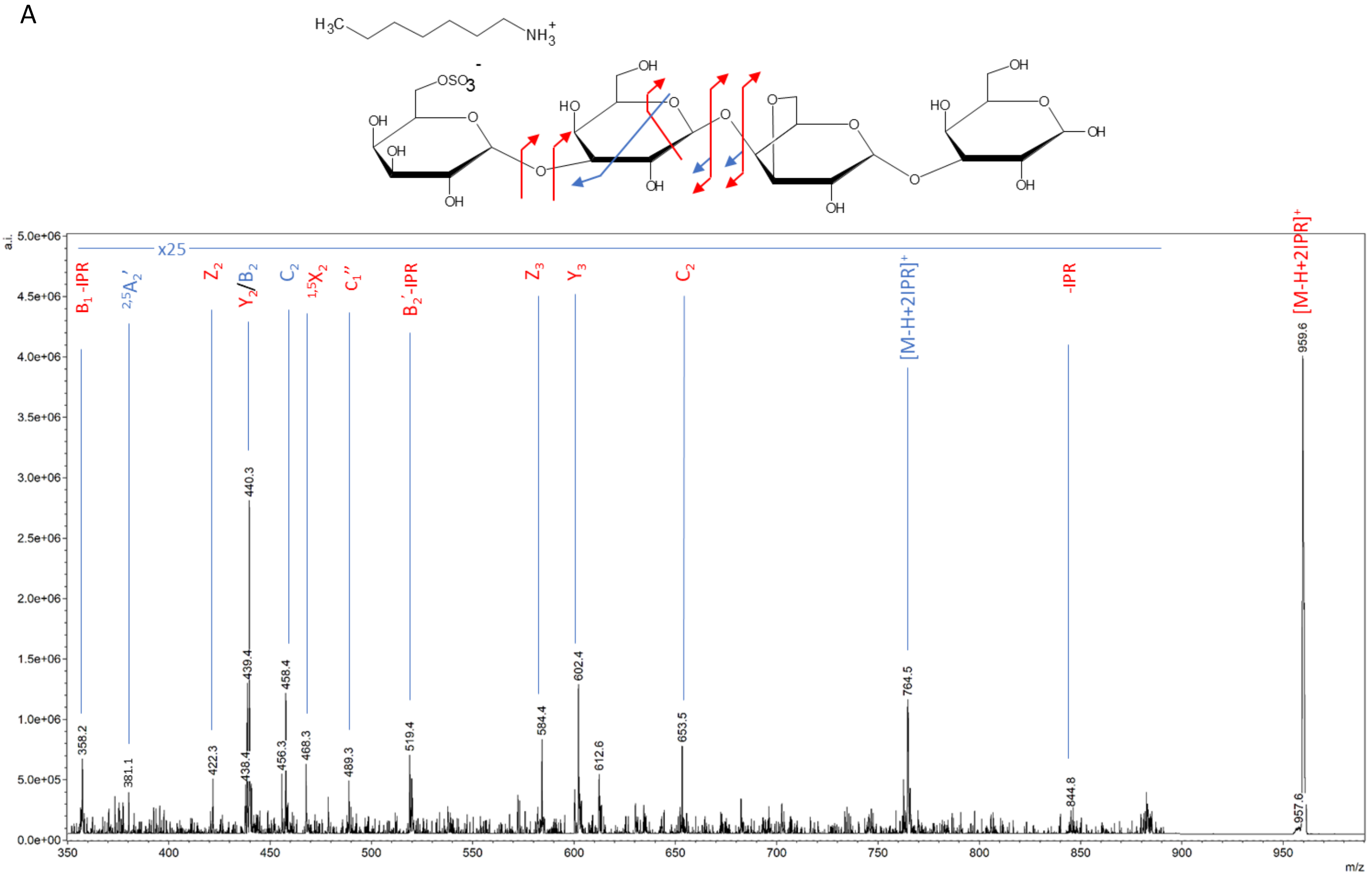

H3C
NH3+
OSO3-
OH
O
HO
OH
x25
B1 -IPR
2,5A2'
Z2
Y2/B2
C2
1,5X2
C1''
B2'-IPR
Z3
Y3
C2
[M-H+2IPR]+
-IPR
[M-H+2IPR]+
a.i.
5.0e+06
4.5e+06
4.0e+06
3.5e+06
3.0e+06
2.5e+06
2.0e+06
1.5e+06
1.0e+06
5.0e+05
0.0e+00
358.2
381.1
422.3
438.4
439.4
440.3
456.3
458.4
468.3
489.3
519.4
584.4
602.4
612.6
653.5
764.5
844.8
957.6
959.6
350
400
450
500
550
600
650
700
750
800
850
900
950
m/z

B

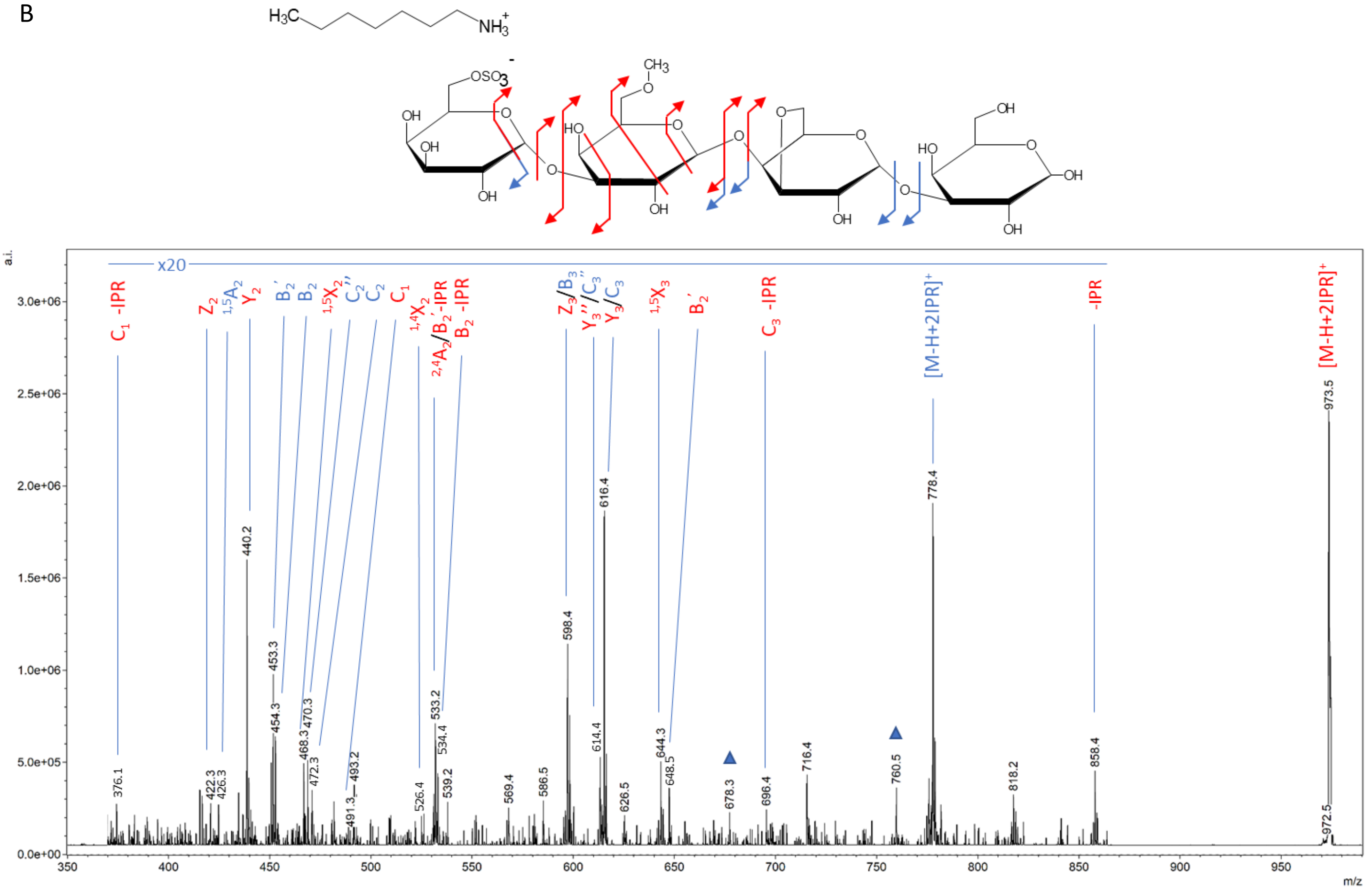

H3C
NH3+
OSO3-
CH3
O
OH
HO
x20
C1 -IPR
Z2
1,5A2
Y2
B2'
B2
1,5X2
C2''
C2
C1
1,4X2
2,4A2/B2'-IPR
B2 -IPR
Z3/B3
Y3''/C3''
Y3/C3
1,5X3
B2'
C3 -IPR
[M-H+2IPR]+
-IPR
[M-H+2IPR]+
a.i.
3.0e+06
2.5e+06
2.0e+06
1.5e+06
1.0e+06
5.0e+05
0.0e+00
376.1
422.3
426.3
440.2
453.3
454.3
468.3
470.3
472.3
491.3
493.2
526.4
533.2
534.4
539.2
569.4
586.5
598.4
614.4
616.4
626.5
644.3
648.5
678.3
696.4
716.4
760.5
778.4
818.2
858.4
972.5
973.5
350
400
450
500
550
600
650
700
750
800
850
900
950
m/z

C

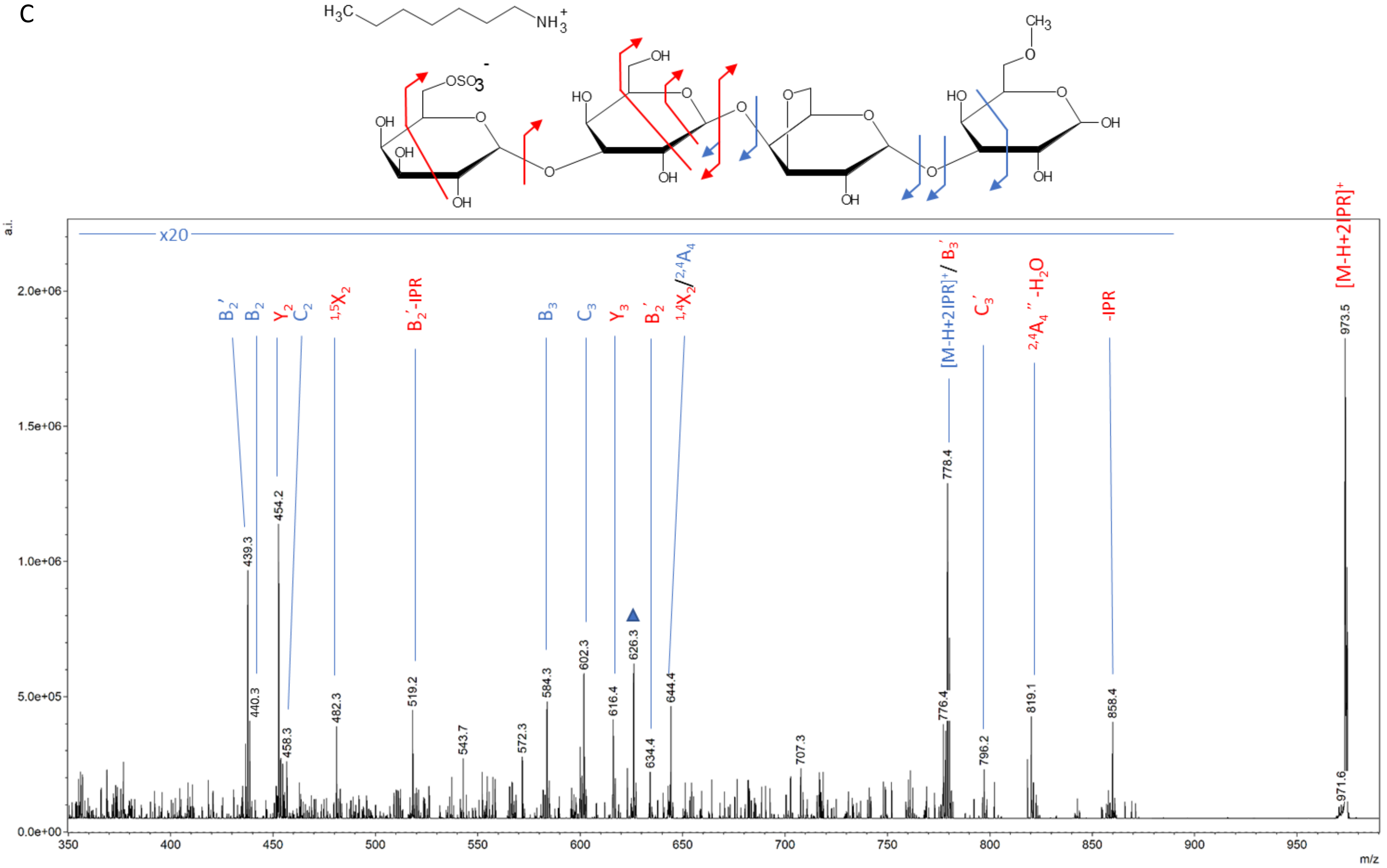

Figure S5. Helium charge transfer dissociation tandem MS spectra of [M-H+2IPR]$^{+}$ precursors at A) *m/z* 959.5 (1.L6S+1.LA+2.G), B) and C) *m/z* 973.5 (1.L6S+1.LA+2.G+1.Me) for two methylated isomers in positive ion mode. The structures of L6S-G-LA-G, L6S-G(Me)-LA-G and L6S-G-LA-G(Me), found in the sample with low MW oligo-porphyrans (F21-22) pretreated with ZgAgaB and digested by CcGH16-3, are annotated with their fragments: red represents fully sulfated fragments, blue, fragments with one sulfate loss and triangle is for a loss of water. A loss of the ion pairing reagent (heptylamine) is indicated as "-IPR" and one IPR (not represented on the schema) gives the charge state of the ion.

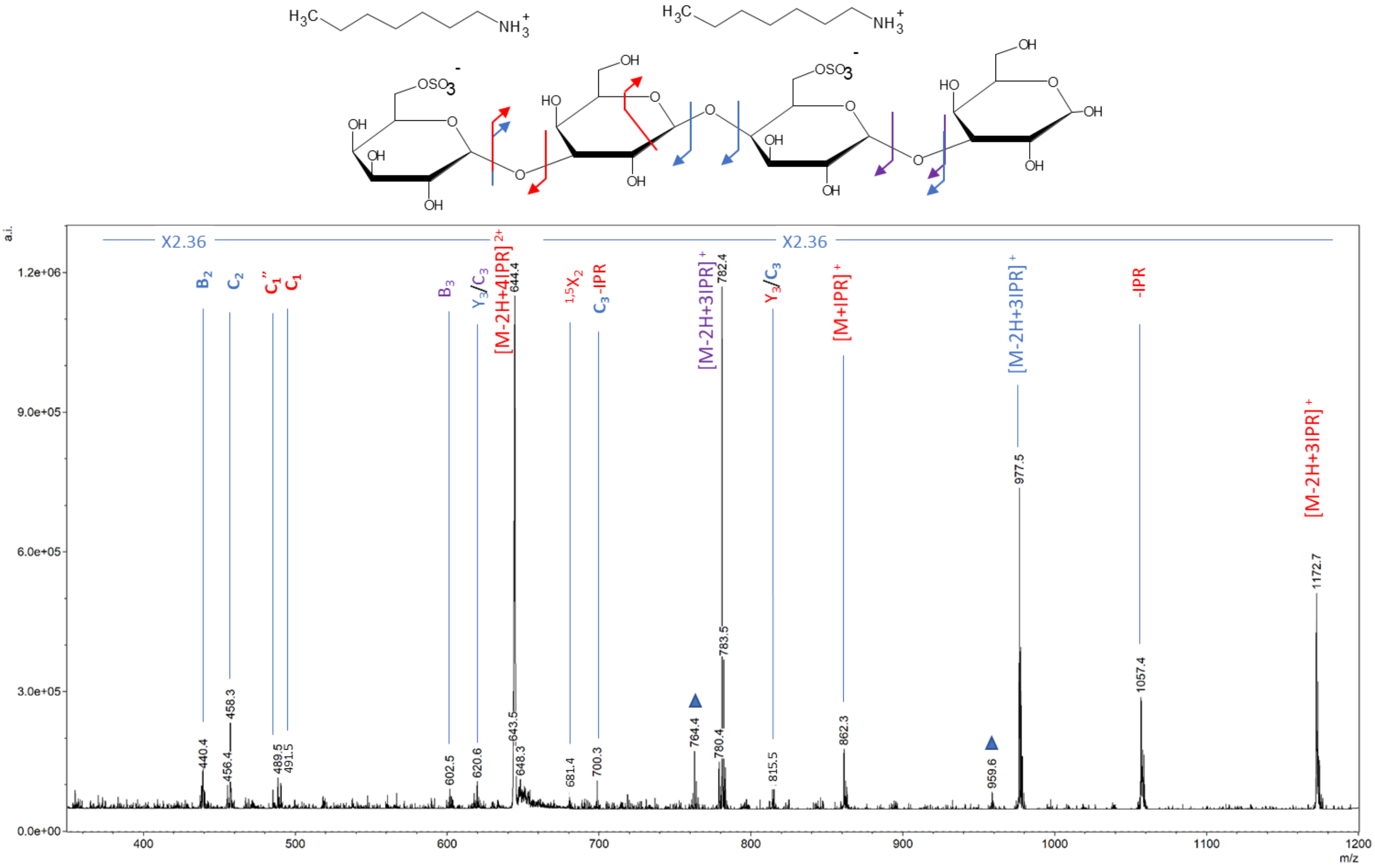

Figure S6. Helium charge transfer dissociation tandem MS spectrum of [M-2H+4IPR]$^{2+}$ precursor at *m/z* 644.3 (2.L6S+2.G) in positive ion mode. The structure of L6S-G-L6S-G, found in the sample with high MW oligo-porphyrans (F5-16) pretreated with ZgAgaB and digested by CcGH16-3, is annotated with their fragments: red represents fully sulfated fragments; blue, fragments with one sulfate loss; purple, fragments with two sulfates losses and triangle is for a loss of water. A loss of the ion pairing reagent (heptylamine) is indicated as "-IPR" and one IPR (not represented on the schema) gives the charge state of the ion.

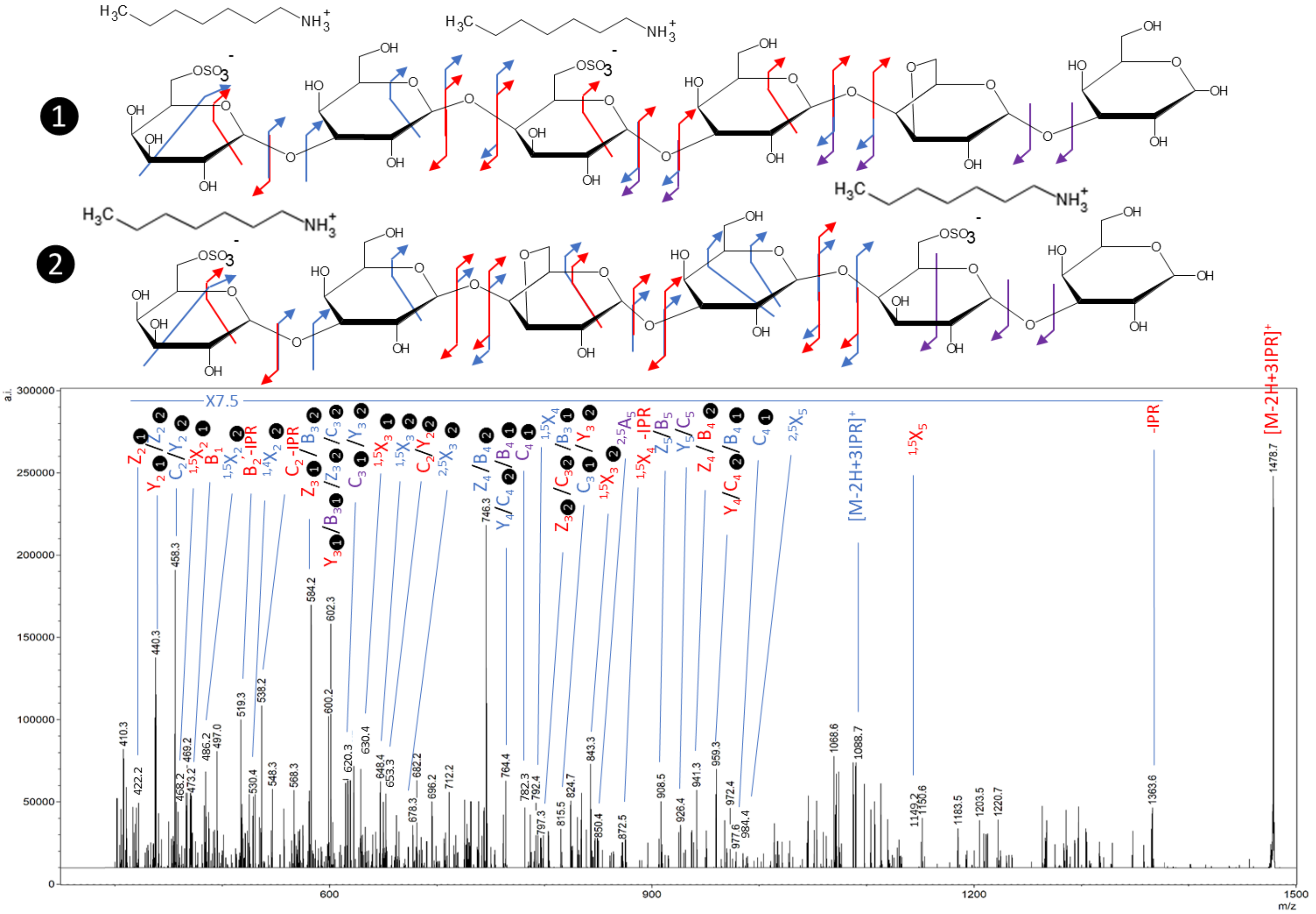

Figure S7. Helium charge transfer dissociation tandem MS spectrum of [M-2H+3IPR]$^{+}$ precursor at *m/z* 1478.6 in positive ion mode. The structures of the two co-eluted isomers (2.L6S+1.LA+3.G), L6S-G-L6S-G-LA-G and L6S-G-LA-G-L6S-G, found in the sample with porphyran polysaccharide digested by CcGH16-3, are annotated with their fragments: red represents fully sulfated fragments; blue, fragments with one sulfate loss and purple, fragments with two sulfates losses. A loss of the ion pairing reagent (heptylamine) is indicated as "-IPR" and one IPR (not represented on the schema) gives the charge state of the ion. The fragments are assigned to the corresponding structure with ❶ and ❷, the unspecified fragments are common for both structures.

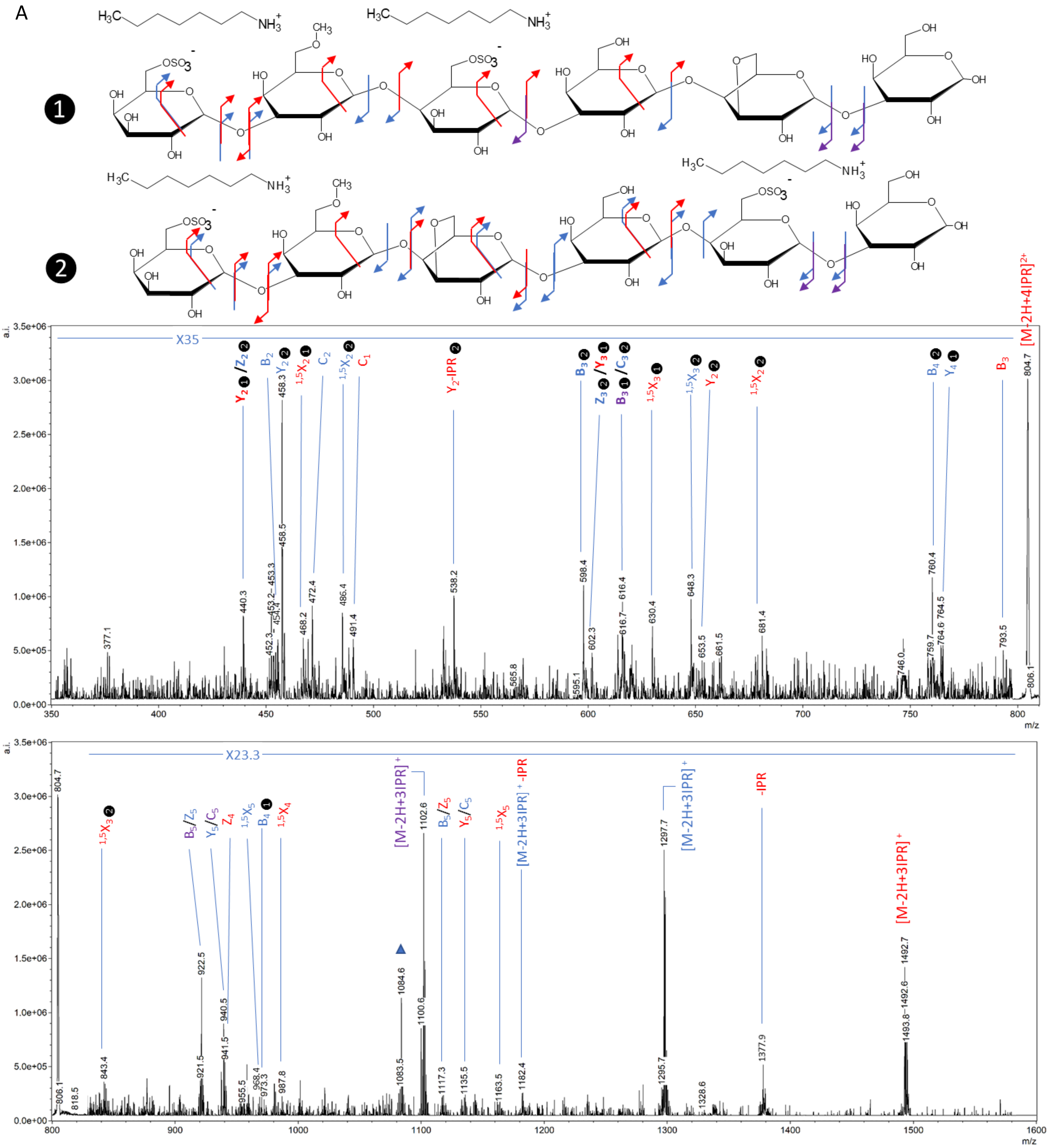

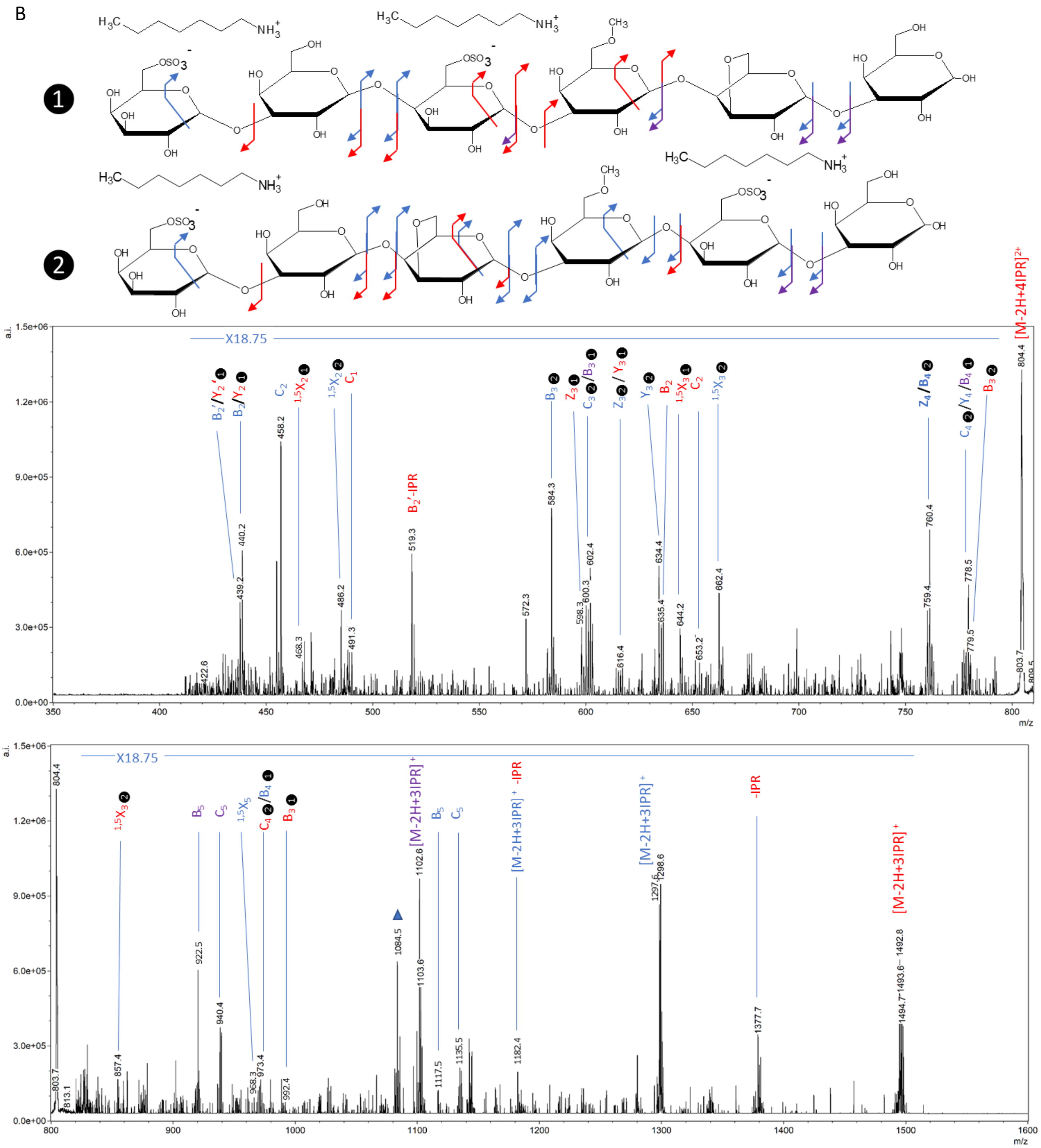

Figure S8. Helium charge transfer dissociation tandem MS spectra of [M-2H+4IPR] $^{2+}$ precursors at *m/z* 804.4 in positive ion mode. There are four methylated DP6 isomers (2.L6S+1.LA+3.G+1.Me), co-eluted by couple, A) L6S-G(Me)-L6S-G-LA-G + L6S-G(Me)-LA-G-L6S-G and B) L6S-G-L6S-G(Me)-LA-G + L6S-G-LA-G (Me)-L6S-G, found in the sample with porphyran polysaccharide digested by CcGH16-3. The structures are annotated with their fragments along the mass range, which is split into two: red represents fully sulfated fragments; blue, fragments with one sulfate loss; purple, fragments with two sulfates losses and a triangle for a loss of water. A loss of the ion pairing reagent (heptylamine) is indicated as “-IPR” and one IPR (not represented on the schema) gives the charge state of the ion. The fragments are assigned to the corresponding structure with ❶ and ❷, the unspecified fragments are common for both structures.

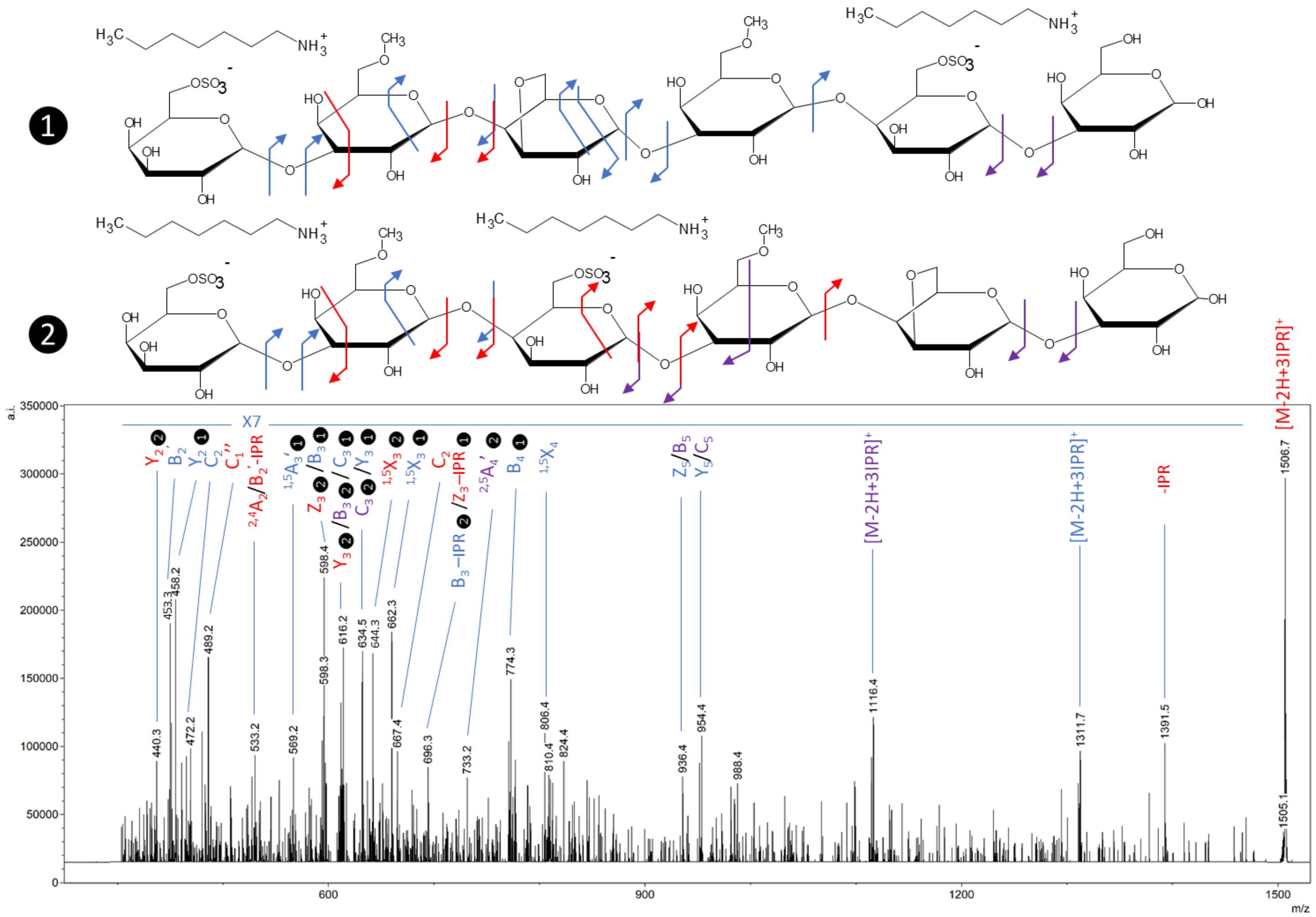

Figure S9. Helium charge transfer dissociation tandem MS spectrum of [M-2H+3IPR]$^+$ precursor at *m/z* 1506.7 (2.L6S+1.LA+3.G+2.Me) in positive ion mode. The structures of L6S-G(Me)-LA-G(Me)-L6S-G and L6S-G(Me)-L6S-G(Me)-LA-G, found in the sample with porphyran polysaccharide digested by GH16, are annotated with their fragments: red represents fully sulfated fragments; blue, fragments with one sulfate loss and purple, fragments with two sulfates losses. A loss of the ion pairing reagent (heptylamine) is indicated as "-IPR" and one IPR (not represented on the schema) gives the charge state of the ion. The fragments are assigned to the corresponding structure with ❶ and ❷, the unspecified fragments are common for both structures.

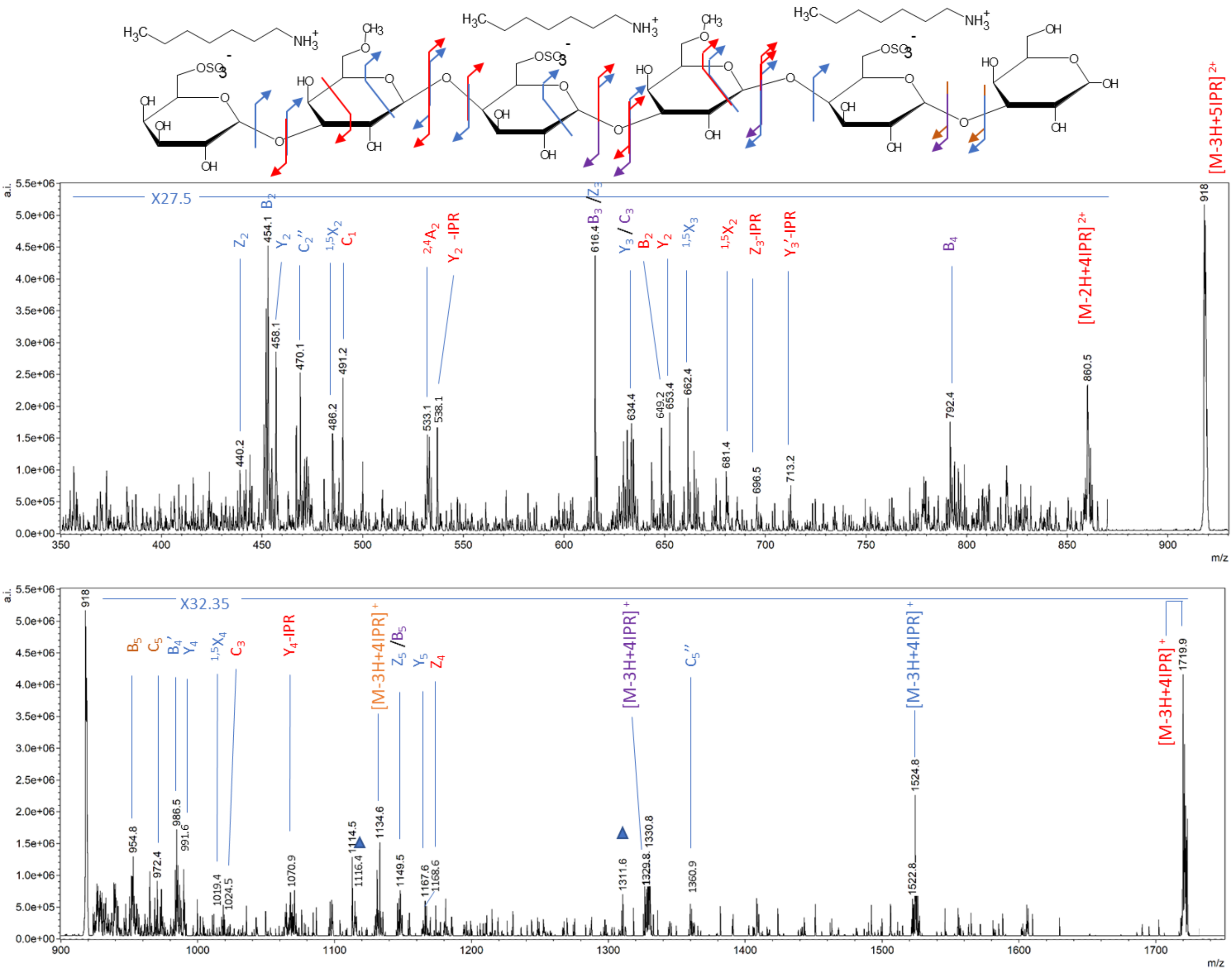

Figure S10. Helium charge transfer dissociation tandem MS spectrum of [M-3H+5IPR]$^{2+}$ precursor at *m/z* 919.4 in positive ion mode. The structure of the DP6 (3.L6S+3.G+2.Me), L6S-G(Me)-L6S-G(Me)-L6S-G, found in the sample with porphyran polysaccharide digested by CcGH16-3, is annotated with their fragments along the mass range, which is split into two: red represents fully sulfated fragments; blue, fragments with one sulfate loss; purple, fragments with two sulfates losses and triangle is for a loss of water. A loss of the ion pairing reagent (heptylamine) is indicated as "-IPR" and one IPR (not represented on the schema) gives the charge state of the ion.

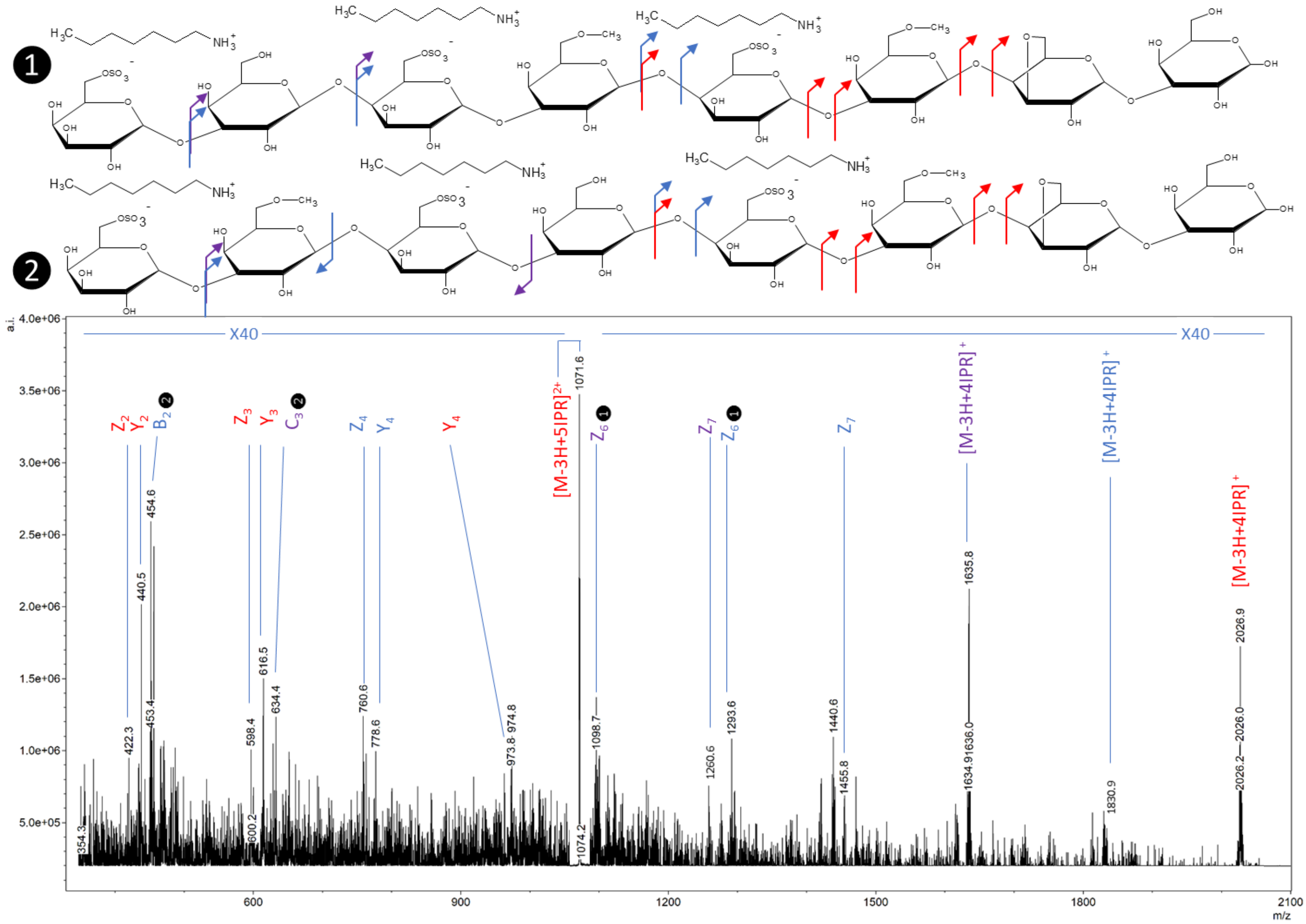

Figure S11. Helium charge transfer dissociation tandem MS spectrum of [M-3H+5IPR] $^{2+}$ precursor at *m/z* 1071 in positive ion mode. The structures of the DP8 (3.L6S+1.LA+4.G+2.Me), L6S-G-L6S-G(Me)-L6S-G(Me)-LA-G and L6S-G(Me)-L6S-G-L6S-G(Me)-LA-G, found in the sample with porphyran polysaccharide digested by CcGH16-3, is annotated with their fragments: red represents fully sulfated fragments; blue, fragments with one sulfate loss and purple, fragments with two sulfates losses. A loss of the ion pairing reagent (heptylamine) is indicated as “-IPR” and one IPR (not represented on the schema) gives the charge state of the ion. The fragments are assigned to the corresponding structure with ❶ and ❷, the unspecified fragments are common for both structures.

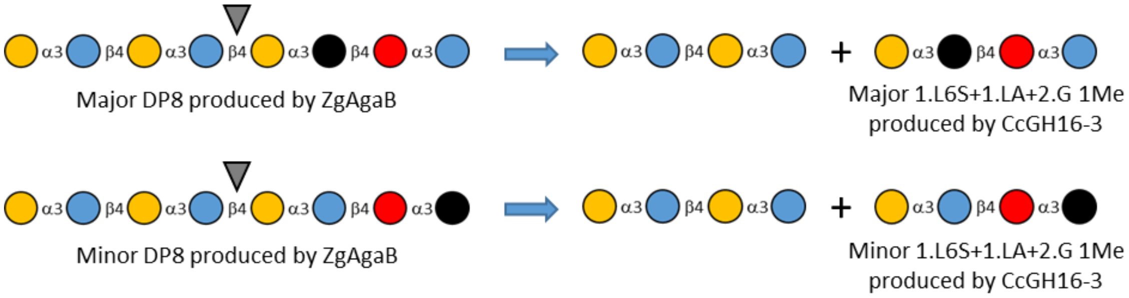

Figure S12. Scheme of the enzymatic reactions highlighted by the structural characterization of the DP8 oligosaccharides in the low MW oligo-porphyrans produced by ZgAgaB (Figure S4) before and after digestion by CcGH16-3 (Figure S5), see also Table 3.

Table S1. Sequence accession numbers of red algal GH16 and representatives of GH16 subfamilies 11,12,13 and 14 used for the structural alignment (Figure S1) and subfamilies 11, 12, 13, 14,15,16,17,26 and outgroup 3 used for the phylogeny (Figure 2). Where available, 3D structures (pdb id) are shown.

organism name	name of enzyme	GenBank acc no.	Pdb id
Chondrus crispus	GH16_xx-Cc-1	CDF40276.1	
Chondrus crispus	GH16_xx-Cc-2	CDF41280.1	
Chondrus crispus	GH16_xx-CcPorA	CDF33251.1	3ilf
Porphyra umbilicalis	GH16_xx-Pu-1	OSX73714.1	
Porphyra umbilicalis	GH16_xx-Pu-2	OSX73300.1	
Porphyra umbilicalis	GH16_xx-Pu-3	OSX79986.1	
Gracilariopsis chorda	GH16_xx-Gc-1	PXF45111.1	
Gracilariopsis chorda	GH16_xx-Gc-2	PXF45120.1	
Gracilariopsis chorda	GH16_xx-Gc-3	PXF47303.1	
Zobellia galactanivorans DsijT	GH16_11-ZgPorB	CAZ95074.1	3juu
Zobellia galactanivorans DsijT	GH16_11-ZgPorC	CAZ97514.1	
Paraglaciecola mesophila GPM4	GH16_11-PmPorB	QHJ13074.1	
Verrucomicrobia bacterium S94	GH16_11-Vb-1	QBG49234.1	
Cellulophaga baltica 18	GH16_11-Cb-1	AIZ43720.1	
Wenyingzhuangia fucanilytica CZ1127	GH16_11-WfPorA	ANW96028.1	
Pseudoalteromonas atlantica T6c	GH16_12-Pa-1	ABG39352.1	
Zobellia galactanivorans DsijT	GH16_12-ZgPorD	CAZ97766.1	
Zobellia galactanivorans DsijT	GH16_12-ZgPorE	CAZ97778.1	
Bacteroides plebeius (Phocaeicola plebeius DSM 17135*)*	GH16_12-BpPorB	EDY95423.1	4awd
Gramella forestii KT0803	GH16_12-Gf-1	CAL66695.1	
Verrucomicrobia bacterium S94	GH16_12-VbPorA	QBG46631.1	
Rhodopirellula baltica	GH16_13-RbCgkA	CAD73010.1	
Paraglaciecola hydrolytica	GH16_13-PhCgbA	WP_082768820.1	
Paraglaciecola hydrolytica	GH16_13-PhCgbB	WP_082768866.1	
Alteromonas sp. KC3	GH16_13-As-1	BCO19660.1	
Pseudoalteromonas carrageenovora KCTC 22325	GH16_13-Pc-1	QBJ74060.1	
Wenyingzhuangia fucanilytica CZ1127	GH16_14-WfPorB	ANW96030.1	
Gramella forsetii KT0803	GH16_14-Gf-2	CAL66698.1	
Flavobacterium faecale	GH16_14-Ff-1	AWG22648.1	
Pseudoalteromonas atlantica T6c	GH16_14-Pa-2	ABG39333.1	
Verrucomicrobia bacterium S94	GH16_14-Vb-2	QBG46180.1	
Zobellia galactanivorans DsijT	GH16_15-ZgAgaC	CAZ98402.1	6hy3
Uncultured bacterium	GH16_15-UkAguF	AAP49324.1	
Catenovulum agarivorans YM01	GH16_15-CaAgaA	AIT14660.1	
Alteromonas sp. GNUM-1	GH16_15-AsAgaG	AGW43026.1	
Tenacibaculum jejuense KCTC 22618	GH16_15-Tj-1	SNR16615.1	
Zobellia galactanivorans DsijT	GH16_16-ZgAgaB	CAZ97711.1	4atf
Saccharophagus degradans 2-40	GH16_16-SdAgaB	ABD80437.1	
Pseudoalteromonas atlantica T6c	GH16_16-PaAgaA	AAA91888.1	
Vibrio sp. PO-303	GH16_16-VsAgaD	BAF34350.1	
Bacteroides plebeius (Phocaeicola plebeius DSM 17135 *)*	GH16_16-BpAgaA	WP_007560915.1	
Zobellia galactanivorans DsijT	GH16_17-ZgCgkA	CAZ94309.1	5ocr
Pseudoalteromonas carrageenovora KCTC 22325	GH16_17-PcCgkA	CAA50624.1	5ocq
Wenyingzhuangia sp. OF219	GH16_17-WsCgkA	AST13124.1	
Rhodopirellula baltica	GH16_17-RbCgkA	CAD72787.1	
Vibrio sp. SY01	GH16_17-Vs-1	QGN18698.1	
Zobellia galactanivorans DsijT	GH16_26-PorA	CAZ96750.1	
Aquimarina sp. AD10	GH16_26-As-1	AXT60490.1	
Gramella forsetii KT0803	GH16_26-Gf-3	CAL66684.1	
Planctomycetes bacterium MalM25	GH16_26-Pb-1	QDT69141.1	
Tenacibaculum jejuense KCTC 22618	GH16_26-Tj-2	SNR17473.1	
Pyrococcus furiosus DSM 3638	GH16_3-PfLamA	AAC25554.2	2vyo
Acetivibrio thermocellus DSM 1237	GH16_3-AtLicA	CAA61884.2	3wvj
Flavobacterium sp. 4221	GH16_3-FsLamA	ABW02990.1	
Cryptopygus antarcticus	GH16_3-CaLamA	ACD93221.1	
Rhodothermus marinus ITI278	GH16_3-RmLicA	AAA60459.1	3iln

Table S2. Primers used for cloning, site directed-mutagenesis and intergenic amplification of *C. crispus* DNA. Restriction sites and modified codons in the primer sequences are in bold.

Primer	***Sequence (5′ →3′)***	***Restriction site***
ccgh16-3-F	AAAAAA**GGATCC**GGGTTTCTCGACAAAATATTCGGTC	*BamHI*
ccgh16-3-R	AAAAAA**GAATTC**TCACGCCTTCTTCCACGTCCGC	*EcoRI*
ccgh16-3-E141Q-F	GCAGGAGGATTGGAAC**CAG**ATCGACGTGTTCGAGT	
ccgh16-3-E141Q-R	ACTCGAACACGTCGAT**CTG**GTTCCAATCCTCCTGC	
ccgh16-3-D143N-F	GGATTGGAACGAGAT**CAA**CGTGTTCGAGTCAAGCC	
ccgh16-3-D143N-R	GGCTTGACTCGAACAC**GTT**GATCTCGTTCCAATCC	
ccgh16-3-E146Q-F	CGAGATCGACGTGTTC**CAG**TCAAGCCAGGCGGCGG	
ccgh16-3-E146Q-R	CCGCCGCCTGGCTTGA**CTG**GAACACGTCGATCTCG	
ccgh16-3-H162Q-F	GTTTCACATGAACATG**CAA**GTGTTCCGCAAGGACG	
ccgh16-3-H162Q-R	CGTCCTTGCGGAACAC**TTG**CATGTTCATGTGAAAC	
chc_t00002034001-F	ATTGGTGCACCAACGACAGG	
genccgh16-3-R	TCCCACTTGGTGGTGTCAAG	

Table S3. Bacterial strains and plasmids used for cloning and protein expression.

Strains or plasmids	***Phenotype***	***Reference***
E. coli Stellar	*F-, endA1, supE44, thi-1, recA1, relA1, gyrA96, phoA, Φ80d lacZΔ M15, Δ(lacZYA-argF) U169, Δ(mrr-hsdRMS-mcrBC), ΔmcrA, λ-*	Takara
E. coli BL21(DE3)	*fhuA2 [lon] ompT gal (λ DE3) [dcm] ΔhsdS λ DE3 = λ sBamHIo ΔEcoRI-B int::(lacI::PlacUV5::T7 gene1) i21 Δnin5*	NEB
Plasmids		
pRF3	pFO4 derivative plasmid, expression vector; Amp^R	This study
pMG1	*ccgh16-3* cloned in pRF3	This study
pMG2	*ccgh16-3*-E141Q mutagenesis from pMG1	This study
pMG3	*ccgh16-3*-D143N mutagenesis from pMG1	This study
pMG4	*ccgh16-3*-E146Q mutagenesis from pMG1	This study
pMG5	*ccgh16-3*-H162Q mutagenesis from pMG1	This study

Table S4. Mass spectrometry-based glycomic identification data for ESI-MS analyses.

Experimental Mass/Charge	Charge observed	Identification	Theoretical mass/charge	Nature of the ion	Deviation between experimental and theoretical mass (m/z)
Figure 8A					
613.12		DP10 3.L6S+2.LA+5.G	613.12	$[M-3.H]^{3-}$	0.00
817.70	2	DP8 3.L6S+1.LA+4.G	817.70	$[M-2.H+HxA]^{2-}$	0.00
824.70	2	DP8 3.L6S+1.LA+4.G+1.Me	824.71	$[M-2.H+HxA]^{2-}$	0.01
831.71	2	DP8 3.L6S+1.LA+4.G+2.Me	831.72	$[M-2.H+HxA]^{2-}$	0.01
Figure 8B					
421.07	1	DP2 1.L6S+1.G	421.07	$[M-H]^{-}$	0.00
565.11	2	DP6 2.L6S+1.LA+3.G	565.11	$[M-2.H]^{2-}$	0.00
745.84	3	DP10 5.L6S+5.G	745.85	$[M-3.H+2.HxA]^{3-}$	0.01
750.51	3	DP10 5.L6S+5.G+1.Me	750.53	$[M-3.H+2.HxA]^{3-}$	0.02
755.19	3	DP10 5.L6S+5.G+2.Me	755.19	$[M-3.H+2.HxA]^{3-}$	0.00
847.88	3	DP12 5.L6S+1.LA+6.G	847.88	$[M-3.H+2.HxA]^{3-}$	0.00
852.55	3	DP12 5.L6S+1.LA+6.G+1.Me	852.55	$[M-3.H+2.HxA]^{3-}$	0.00
857.22	3	DP12 5.L6S+1.LA+6.G+2.Me	857.22	$[M-3.H+2.HxA]^{3-}$	0.00
861.89	3	DP12 5.L6S+1.LA+6.G+3.Me	861.89	$[M-3.H+2.HxA]^{3-}$	0.00
914.24	3	DP12 6.L6S+6.G	914.24	$[M-3.H+3.HxA]^{3-}$	0.00
918.91	3	DP12 6.L6S+6.G+1.Me	918.91	$[M-3.H+3.HxA]^{3-}$	0.00
923.57	3	DP12 6.L6S+6.G+2.Me	923.58	$[M-3.H+3.HxA]^{3-}$	0.01
949.91	3	DP14 5.L6S+2.LA+7.G	949.91	$[M-3.H+2.HxA]^{3-}$	0.00
954.58	3	DP14 5.L6S+2.LA+7.G+1.Me	954.58	$[M-3.H+2.HxA]^{3-}$	0.00
1016.27	3	DP14 6.L6S+1.LA+7.G	1016.27	$[M-3.H+3.HxA]^{3-}$	0.00
1020.94	3	DP14 6.L6S+1.LA+7.G+1.Me	1020.95	$[M-3.H+3.HxA]^{3-}$	0.01
1025.61	3	DP14 6.L6S+1.LA+7.G+2.Me	1025.62	$[M-3.H+3.HxA]^{3-}$	0.01
1030.28	3	DP14 6.L6S+1.LA+7.G+3.Me	1030.29	$[M-3.H+3.HxA]^{3-}$	0.01
1184.67		DP16 7.L6S+1.LA+8.G	1184.67	$[M-3.H+4.HxA]^{3-}$	0.00
Figure 8C					
419.07	2	DP4 2.L6S+2.G+1.Me	419.07	$[M-2.H]^{2-}$	0.00
421.06	1	DP2 1.L6S+1.G	421.07	$[M-H]^{-}$	0.01
565.11	2	DP6 2.L6S+1.LA+3.G	565.11	$[M-2.H]^{2-}$	0.00
572.11	2	DP6 2.L6S+1.LA+3.G+1.Me	572.12	$[M-2.H]^{2-}$	0.01
579.12	2	DP6 2.L6S+1.LA+3.G+2.Me	579.12	$[M-2.H]^{2-}$	0.00
727.16	1	DP4 1.L6S+1.LA+2.G	727.16	$[M-H]^{-}$	0.00
741.17	1	DP4 1.L6S+1.LA+2.G+1.Me	741.18	$[M-H]^{-}$	0.01
Figure 8D					
412.06	2	DP4 2.L6S+2.G	412.06	$[M-2.H]^{2-}$	0.00
419.07	2	DP4 2.L6S+2.G+1.Me	419.07	$[M-2.H]^{2-}$	0.00
421.07	1	DP2 1.L6S+1.G	421.07	$M-H]^{-}$	0.00
565.11	2	DP6 2.L6S+1.LA+3.G	565.11	$[M-2.H]^{2-}$	0.00
572.11	2	DP6 2.L6S+1.LA+3.G+1.Me	572.12	$[M-2.H]^{2-}$	0.01
671.65	2	DP6 3.L6S+3.G+1.Me	671.66	$[M-2.H+HxA]^{2-}$	0.01
678.66	2	DP6 3.L6S+3.G+2.Me	678.67	$[M-2.H+HxA]^{2-}$	0.01
727.16	1	DP4 1.L6S+1.LA+2.G	727.16	$[M-H]^{-}$	0.00
741.17	1	DP4 1.L6S+1.LA+2.G+1.Me	741.18	$[M-H]^{-}$	0.01
Figure 8E					
419.07	2	DP4 2.L6S+2.G+1.Me	419.07	$[M-2.H]^{2-}$	0.00
421.06	1	DP2 1.L6S+1.G	421.07	$M-H]^{-}$	0.01
565.10	2	DP6 2.L6S+1.LA+3.G	565.11	$[M-2.H]^{2-}$	0.01
572.11	2	DP6 2.L6S+1.LA+3.G+1.Me	572.12	$[M-2.H]^{2-}$	0.01
579.12	2	DP6 2.L6S+1.LA+3.G+2.Me	579.12	$[M-2.H]^{2-}$	0.00
678.66	2	DP6 3.L6S+3.G+2.Me	678.67	$[M-2.H+HxA]^{2-}$	0.01
831.71	2	DP8 3.L6S+1.LA+4.G+2.Me	831.72	$[M-2.H+HxA]^{2-}$	0.01
838.72	2	DP8 3.L6S+1.LA+4.G+3.Me	838.72	$[M-2.H+HxA]^{2-}$	0.00
1091.31	2	DP10 4.L6S+1.LA +5.G+3.Me	1091.31	$[M-2.H+2.HxA]^{2-}$	0.00
1098.31	2	DP10 4.L6S+1.LA +5.G+4.Me	1098.32	$[M-2.H+2.HxA]^{2-}$	0.01
